# Radiation-resistant metal-organic framework enables efficient separation of krypton fission gas from spent nuclear fuel

**DOI:** 10.1038/s41467-020-16647-1

**Published:** 2020-06-18

**Authors:** Sameh K. Elsaidi, Mona H. Mohamed, Ahmed S. Helal, Mitchell Galanek, Tony Pham, Shanelle Suepaul, Brian Space, David Hopkinson, Praveen K. Thallapally, Ju Li

**Affiliations:** 1 Department of Chemical and Biomolecular Engineering, University of California, Berkeley, Berkeley, CA 94720 USA; 2 Oak Ridge Institute for Science and Education, Pittsburgh, PA 15236 USA; 3 DOE National Energy and Technology Laboratory (NETL), Pittsburgh, PA 15236 USA; 4 Department of Chemistry, University of Pittsburgh, 219 Parkman Avenue, Pittsburgh, USA; 5 Chemistry Department, Faculty of Science, Alexandria University, P.O. Box 426, Ibrahimia, Alexandria, 21321 Egypt; 6 Nuclear Materials Authority, P.O. Box 540, El Maadi, Cairo, Egypt; 7 Department of Nuclear Science and Engineering and Department of Materials Science and Engineering, Massachusetts Institute of Technology, Cambridge, MA 02139 USA; 8 Office of Environment, Health & Safety, Massachusetts Institute of Technology, Cambridge, MA 02139 USA; 9 Department of Chemistry, Biochemistry, and Physics, The University of Tampa, 401 West Kennedy Boulevard, Tampa, FL 33606-1490 USA; 10 Department of Chemistry, University of South Florida, 4202 East Fowler Avenue, CHE205, Tampa, FL 33620 USA; 11 Physical and Computational Science Directorate, Pacific Northwest National Laboratory, Richland, WA 99352 USA

**Keywords:** Chemistry, Energy science and technology, Materials science

## Abstract

Capture and storage of volatile radionuclides that result from processing of used nuclear fuel is a major challenge. Solid adsorbents, in particular ultra-microporous metal-organic frameworks, could be effective in capturing these volatile radionuclides, including ^85^Kr. However, metal-organic frameworks are found to have higher affinity for xenon than for krypton, and have comparable affinity for Kr and N_2_. Also, the adsorbent needs to have high radiation stability. To address these challenges, here we evaluate a series of ultra-microporous metal-organic frameworks, SIFSIX-3-M (M = Zn, Cu, Ni, Co, or Fe) for their capability in ^85^Kr separation and storage using a two-bed breakthrough method. These materials were found to have higher Kr/N_2_ selectivity than current benchmark materials, which leads to a notable decrease in the nuclear waste volume. The materials were systematically studied for gamma and beta irradiation stability, and SIFSIX-3-Cu is found to be the most radiation resistant.

## Abstract (web summary)

Management of spent nuclear fuel is challenging due to the release of volatile radionuclides. Here the authors report krypton separation from fission gas in the presence of other competing gases by a radiation resistant metal-organic framework using the two-bed breakthrough technique.

## Introduction

Nuclear energy is an emission free, high-energy density source with minimal land use. However, any future expansion of civilian nuclear power will most likely require efficient management of used nuclear fuel (UNF). UNF processing minimizes radioactive waste, but the release of volatile radionuclides is a significant challenge. The nature of the volatile radionuclides depends on the reprocessing procedure and generally consists of a mixture of noble gases (predominantly ^85^Kr, which β or βγ decays to stable ^85^Rb with *t*_1/2_ = 10.8 years, along with Xe), and species containing ^129^I^1–5^. The current reprocessing technologies can capture other volatile radionuclides with relative ease, but an efficient system to capture (and store) ^85^Kr needs to be in place. While methods such as cryogenic distillation and fluorocarbon based absorption have been proposed and tested, they are expensive and require complex engineering control. Solid-state adsorbents, in particular porous metal-organic frameworks (MOFs), could be better alternatives to capture these volatile radionuclides including ^85^Kr.

Physisorption-based adsorption and storage is deemed an energy-efficient process that can be operated at near-ambient conditions and is easier to integrate into current engineering setups. Several traditional adsorbents such as zeolites and activated carbon have been tested, but were found to have low capacity and selectivity (over Xe and other competing gases). MOFs are known for their versatile architecture and functionalized pore surface and have shown promise for gas sorption and separation^1,6–16^. However, as Xe is considerably more polarizable than Kr, porous materials such as MOFs are generally more selective toward Xe over Kr due to stronger van der Waals interactions, which causes further engineering challenges as the Xe will lead to reduced Kr adsorption^2,7,15,17–19^. To avoid this, we recently reported a proof-of-concept study where a dual-bed system, fitted in series, was utilized to separate and store Kr^20^. The gas stream (known as off-gas, 400 ppm of Xe and 40 ppm of Kr mixed with air, which are the typical concentrations of the off-gas in a reprocessing plant) is first directed through a Xe selective adsorbent bed to remove the Xe, followed by removal of Kr in the second bed using the same or another adsorbent material. In the absence of the competing Xe in the second bed, the adsorbent is expected to have enhanced Kr storage capacity, even when using identical adsorbent material (which is the modality used in this paper). The enhancement and the total Kr uptake depend on the selectivity for Kr over competing gases (e.g., N_2_, O_2_). The stored gas in the second bed has a high Kr, low Xe feature, so the second bed can be fluidized and/or regenerated (with temperature controlled desorption) with such characteristics in mind. The MOFs in both the first bed and the second bed should be sufficiently radiation resistant to beta and gamma radiations as ^85^Kr flows pass or stores in them. Only a few MOFs have been studied for their radiation stability^20,21^. Lee et al. reported the potential of three MOFs (MIL-100(Fe), MIL-101(Cr), and UiO-66(Zr)) for Xe/Kr separation^22^. The study showed that UiO-66(Zr) is the most promising adsorbent among the three candidates; however, the radiation stability of UiO-66(Zr) has been performed under low radiation dose of only 2 kGy which is not relevant to the practical Xe/Kr separation at nuclear reprocessing plants.

**Fig. 1:**
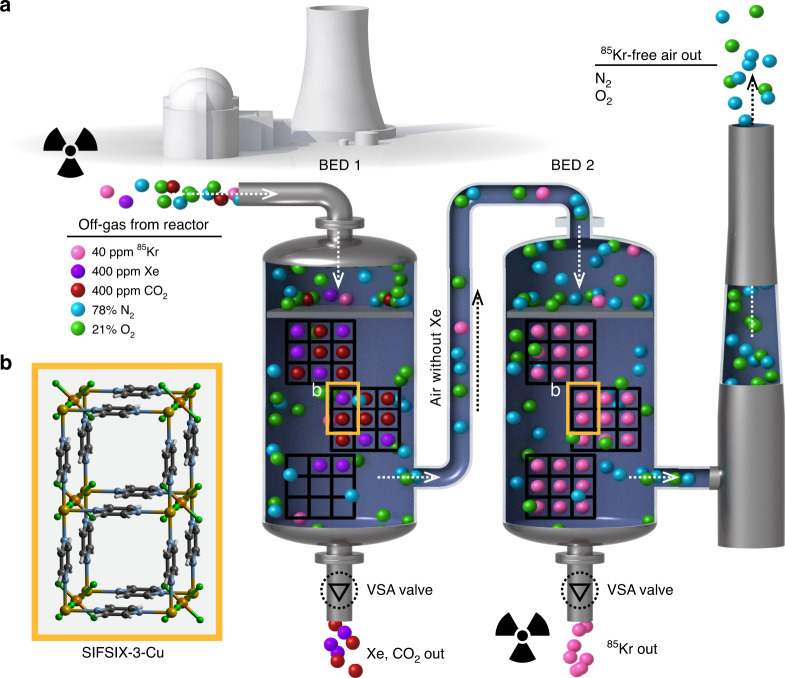
Schematic representation of the two-bed breakthrough setup. **a** Illustration of the two-bed technique for removal of radioactive Kr from nuclear reprocessing plants using SIFSIX-3-Cu. **b** Each two squares in the bed 1 and bed 2 represent two channels of SIFSIX-3-Cu structure. Atom colors: C = gray, H = white, N = light blue, F = green, Si = light brown, Cu = orange, O = red, Xe = violet, Kr = pink.

In our continuous search for materials with high Kr adsorption capacity and selectivity, we synthesized, measured and analyzed the SIFSIX-3-M series (M = Zn, Cu, Ni, Co, Fe) of MOFs^7,23–25^. SIFSIX-3-M is a class of isoreticular hybrid ultra-microporous materials (HUMs) based on saturated metal centers (SMCs) and SiF_6_^−2^ pillars^26,27^. SIFSIX-3-M can be tuned for Kr separation by substituting different metal centers including Zn^27,28^, Co^29^, Cu^30^, Ni^29^, or Fe^8^. SIFSIX-3-M materials are known for their very low affinity toward N_2_ and for exceptional performance in selectively removing CO_2_ from air, which makes them great candidates for two-bed breakthrough setup. In this study, two columns were filled by adsorbent material that has a preferential adsorption of gases Xe, CO_2_ > Kr > N_2_ and O_2_. The Xe and CO_2_ gases will be selectively adsorbed over the rest of gases in the first bed while the Kr will be preferentially adsorbed over the N_2_ and O_2_ in the second bed. The advantage of this method is that we can separate the radioactive ^85^Kr into a high purity gas. The presence of other gases mixed with ^85^Kr would otherwise increase the waste volume to be disposed and therefore increase the cost of the UNF reprocessing (Fig. 1).

## Results

### Radiation stability study

The MOF materials were exposed to gamma radiation (^60^Co source) from 0 to 200 kGy and the stability of the materials was monitored by powder X-ray diffraction (PXRD) to confirm the retention of their crystallinity after γ irradiation (see Methods section for experimental details) and β irradiation. The near equivalence in radiation damage tolerance dose between ^85^Kr β and ^60^Co γ-rays was well established, for example “no significant difference in the decomposition yield was observed” in fluorocarbons exposed to beta and gamma radiation of the same dose^31^.

**Fig. 2:**
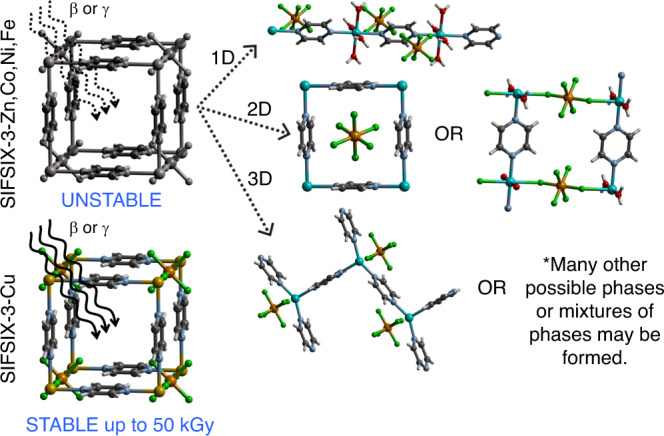
Illustration of impact of irradiation on SIFSIX-3-M materials. The SIFSIX-3-M (M = Zn, Co, Ni, Fe) structures decompose to 1D, 2D or many other possible phases up on radiation exposure. The SIFSIX-3-Cu is stable and maintains its original crystal structure up to 50 kGy dose in beta or gamma radiation. Atom colors: C = gray, H = white, N = light blue, F = green, Si = light brown, Cu = orange, M (Ni, Co, Fe, Zn) = cyan, O = red.

**Fig. 3:**
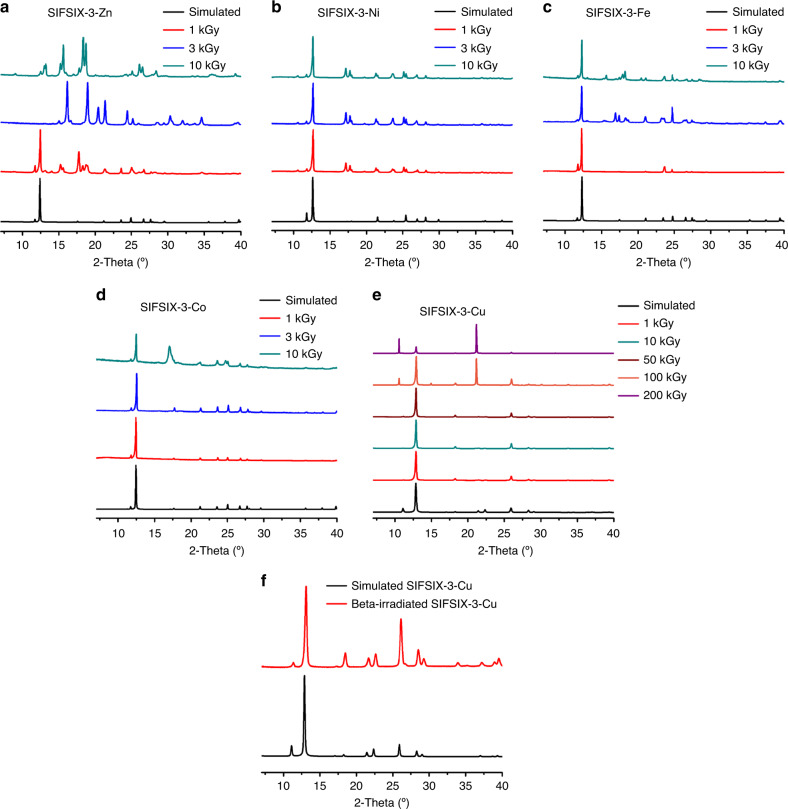
Powder X-ray diffraction patterns of irradiated and simulated SIFSIX-3-M structures. **a** gamma-irradiated SIFSIX-3-Zn, **b** gamma-irradiated SIFSIX-3-Ni, **c** gamma-irradiated SIFSIX-3-Fe, **d** gamma-irradiated SIFSIX-3-Co, and **e** SIFSIX-3-Cu and **f** activated SIFSIX-3-Cu structure after 50 kGy beta irradiation.

Quantitative analyses of the PXRD data using the Rietveld and Pawley fitting methods were performed to compare the as-synthesized structures with the irradiated structures (Supplementary Figs. 3–17). As shown in Figs. 2 and 3 and Supplementary Figs. 3 and 4, the Zn and Ni analogs are unstable and show phase change at 1 kGy. SIFSIX-3-Fe maintained its crystallinity at 1 kGy. Further irradiation of SIFSIX-3-Fe to 3 and 10 kGy leads to new unknown phases as shown in Supplementary Figs. 4–8. The new phase is a mixture of a 1D structure with water coordinated to Fe (Fig. 2) and another unknown phase. The original structure of SIFSIX-3-Fe is strongly distorted and almost vanished. The SIFSIX-3-Co structure was stable up to 10 kGy before it undergoes phase change (Supplementary Figs. 9–12). From the PXRD pattern of irradiated MOF at 3 kGy, higher peak intensity for the (110) peak at 17.65° was observed which may be attributed to spinning of fluorine atoms into the (110) plane (Supplementary Fig. 11). Based on these results the crystal structure of SIFSIX-3-Co remains unaltered under 3 kGy radiation. After 10 kGy radiation, the crystal structure is altered and presents the original SIFSIX-3-Co structure as well as another unknown phase. The unit cell and space group are determined from the unknown phase peak positions (Supplementary Fig. 12) to be tetragonal (4/m) with lattice parameters *a* = 16.086 Å and *c* = 12.952 Å, which does not exist in the CCDC database^32^.

SIFSIX-3-Cu was the most stable MOF (Supplementary Figs. 13–17) and it maintained its crystallinity up to 50 kGy γ irradiation. According to Cambridge Structural Database (CSD)^33^, Cu tends to bond with N donor ligand more readily compared with Co, Fe, Zn, and Ni. The Cu–N bonds are stronger than those of other M–N analogs. This is supported by shorter Cu–N bond distance of 1.9 Å in the SIFSIX-3-Cu compared with the other the M–N analogs (2.1 Å). Also, it was reported that the SIFSIX-3-Cu has a slightly smaller unit cell of 378 Å^3^ versus 388 Å^3^ (SIFSIX-3-Zn), where the authors attributed this observation to the relatively stronger bonding between the Cu(II) and the pyrazine^30^. Therefore, M–N bond strength could be the main factor behind the stability of SIFSIX-3-Cu framework which reaches up to 50 kGy, while other materials demonstrate much lower stability that reaches to maximum 10 kGy (in case of Co). For this class of the isoreticular SIFSIX-3-M structures, it was reported that the change in metal center or the environment around it could lead to different properties of the MOF material^8,21,29,30,34^.

The beta radiation stability of the SIFSIX-3-Cu was evaluated by irradiating the activated sample with 1.5 MeV electrons beam at a dose rate of 50 kGy/h and comparing its PXRD pattern with the simulated SIFSIX-3-Cu pattern. Figure 3f showed that the activated SIFSIX-3-Cu sample is stable after 50 kGy beta irradiation. ^85^Kr concentration is reported to be 1130–1800 TBq/Mg (3510–48,600 Ci/Mg) of spent fuel^35^. Based on these concentrations, the radiation dose rate to 1 g SIFSIX-3-Cu from absorbing all the ^85^Kr in 1 g of spent nuclear fuel was calculated (see Supplementary Note 3). According to the obtained results from both beta and gamma irradiation experiments, SIFSIX-3-Cu is radiation resistant up to 50 kGy. Hence, 1 g of SIFSIX-3-Cu can separate ^85^Kr effectively from 2674 g of spent nuclear fuel (130 TBq/Mg case) or 188 g spent nuclear fuel (1800 TBq/Mg case), without any crystal structure damage, if keeping all the ^85^Kr inside for 1 h.

### Single-component gas adsorption study

**Fig. 4:**
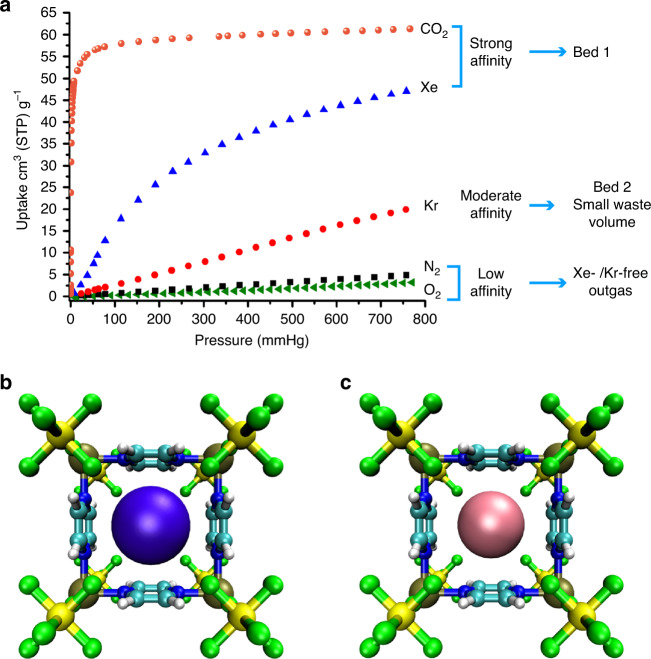
Gas adsorption properties and binding sites. **a** Single-component CO_2_, Xe, Kr, N_2_, and O_2_ adsorption isotherms at 298 K for SIFSIX-3-Cu. Molecular illustration of the most favorable binding site for **b** Xe and **c** Kr in SIFSIX-3-Cu (top view) as determined from simulated annealing calculations. Atom colors: C = cyan, H = white, N = blue, F = green, Si = yellow, Cu = gold, Xe = violet, Kr = pink.

The single-component CO_2_, Xe, Kr, N_2_, and O_2_ adsorption isotherms of SIFSIX-3-Cu at 298 K (Fig. 4a) showed a preferential adsorption of CO_2_ and Xe over Kr which will allow CO_2_ and Xe capture in the first bed, and a high Kr/N_2_ selectivity which facilitates the capture of the ^85^Kr in the second bed.

### Modeling study

As revealed through modeling studies, the larger atomic radius for Xe provided for a better fit within the square pores as well as the region enclosed by four neighboring SiF_6_^2–^ pillars in the material (Fig. 4b, c and Supplementary Figs. 23 and 24). Furthermore, density functional theory (DFT) calculations confirm that SIFSIX-3-Cu is selective for CO_2_ and Xe over Kr, and Kr over N_2_ and O_2_ on the basis of the DFT calculated adsorption energies in the material (see Supplementary Note 5 and Supplementary Table 1).

### Two-bed breakthrough adsorption study

**Fig. 5:**
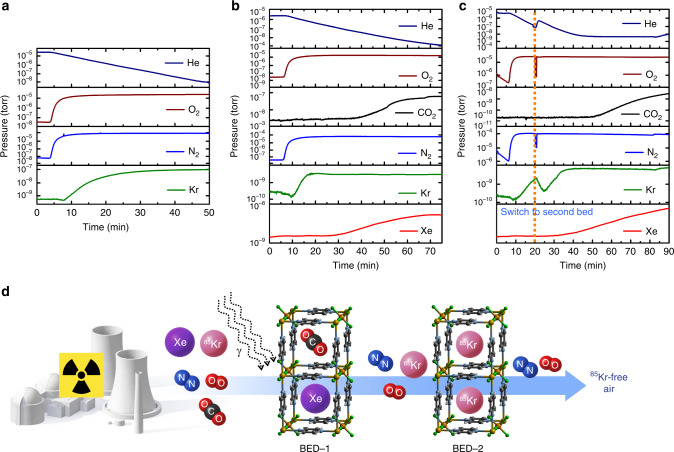
Breakthrough curves for SIFSIX-3-Cu. **a** Single-bed breakthrough experiment using 1000 ppm Kr balanced with dry air. **b** Single-bed breakthrough experiment using 400 ppm Xe and 40 ppm Kr balanced with dry air. **c** Two-bed breakthrough experiment using 400 ppm Xe and 40 ppm Kr balanced with dry air. **d** schematic demonstration of the concept of the two-bed technique for the ^85^Kr removal from the UNF.

We ran the two-bed breakthrough experiments, where both beds use SIFSIX-3-Cu, because of its high radiation stability (Fig. 3e). First, a single-bed test was performed on SIFSIX-3-Cu (Fig. 5a, b) in order to show the ability of SIFSIX-3-Cu for separation of Kr gas from N_2_ and O_2_. Second, single-bed experiments were run for the simulated off-gas containing 400 ppm Xe and 40 ppm Kr balanced with dry air, revealing the time needed for each gas to break through from the first bed under the conditions of simulated off-gas stream. Accordingly, this information informed us of when to switch on the second bed in the two-bed system before Xe and CO_2_ start to break through (see Fig. 1, Supplementary Note 6 and Supplementary Scheme 1). The single-bed breakthrough experiments for 1000 ppm Kr balanced with dry air revealed that SIFSIX-3-Cu can selectively adsorb Kr over N_2_ and O_2_ as shown in Fig. 5 and Table 1.

**Table 1:** Xe and Kr separation performance parameters for SIFSIX-3-Cu at 298 K and 1 bar.

Material	SIFSIX-3-Cu
Xe capacity^a^ (mmol/kg)	6.74
Kr capacity^b^ (mmol/kg)	0.14
Kr capacity^c^ (mmol/kg)	0.15
Kr capacity^d^ (mmol/kg)	6.35
Xe/Kr selectivity^e^	4.81
Kr/N_2_ selectivity^f^	24.38

Gas mixture 1: 400 ppm Xe, 40 ppm Kr, balanced with dry air

^a^Xe capacity at equilibrium in bed 1 for gas mixture 1.

^c^Kr capacity at equilibrium in bed 2 for gas mixture 1.

^e^Xe/Kr selectivity at equilibrium for bed 1.

Gas mixture 2: 1000 ppm Kr, balanced with dry air

^b^Kr capacity at equilibrium in bed 1 for gas mixture 1.

^d^Kr capacity at equilibrium for gas mixture 2.

^f^Kr/N_2_ selectivity at equilibrium for gas mixture 2.

Based on the single-column experiment, SIFSIX-3-Cu can selectively separate Xe from gas mixture 1 consisting of 400 ppm Xe, 40 ppm Kr balanced with dry air. The column breakthrough experiment suggests that Xe breakthrough occurs at *t*_1_^finish^ = 29 min, thus by connecting a second bed loaded with adsorbent material before this time, we will be able to capture the Kr in the second bed before the Xe breaks through the first bed (see Fig. 5). We note that *t*_1_^finish^ certainly depends on the size and geometric design of the first bed (which can be fluidized^36^), i.e., it is not just a material property.

The two-column breakthrough system consists of two adsorption beds in series that were packed with 1 g of SIFISIX-3-Cu in each bed. The adsorbent beds were purged with He and then a gas mixture containing 400 ppm Xe and 40 ppm Kr balanced with dry air at a total pressure of 1 bar was introduced to the first bed (with the second bed bypassed). Breakthrough curves indicate that the Xe gas and CO_2_ were retained by the first bed, leaving 40 ppm Kr balanced with dry air at the outlet. At time *t*_2_^start^ = 18 min, the second bed was enabled, thus flowing gas from the first bed to the second. Gas analysis shows that Kr concentration drops as it is adsorbed into the second bed, and fully breaks through again at *t*_2_^finish^ = around 30 min. In practice the feed gas would be directed to a new bed, while the Kr loaded bed is regenerated. It is important to note that the adsorbent material can capture Kr gas over the competing gases that could increase the waste volume. The benchmark materials, SBMOF-1^37^, Ni-MOF-74^38^, and Ag mordenite^39^, showed remarkable performance for Xe/Kr separation, however, they are not suitable for ^85^Kr separation from spent fuel using the two-bed technique. Ni-MOF-74^38^ and Ag mordenite^39^ were found to have poor Kr/N_2_ selectivity (Supplementary Figs. 21 and 30), while, SBMOF-1^37^ showed low Kr/CO_2_ which prohibit the separation of the Kr in pure form (Supplementary Fig. 22).

As shown in Fig. 5 and Table 1, SIFISIX-3-Cu showed a great potential for Kr removal from simulated UNF off-gas using the two-bed system. The calculated capacity of the adsorbed Xe in first bed by SIFSIX-3-Cu at equilibrium was found to be 6.74 mmol/kg while the capacity of the Kr captured in second bed is 0.15 mmol/kg. This system is a feasible and efficient way to separate and capture Xe and Kr at ambient conditions. This further demonstrates that once the competing gas, Xe, is removed in the first bed using SIFSIX-3-Cu, the Kr removal efficiency was increased significantly, which can lead to a notable decrease in the waste volume at the UNF reprocessing plant.

## Discussion

In summary, we evaluated the performance of a family of ultra-microporous pillared square lattices, SIFSIX-3-M (M = Fe, Co, Ni, Cu, Zn) for ^85^Kr removal from used nuclear fuel. The radiation stability was examined by exposing the materials to varying levels of gamma radiation and beta radiation.

SIFSIX-3-Cu is the only material in this family suitable for this application based on radiation stability up to 50 kGy for both beta and gamma radiations.

The choice of the right material for the ^85^Kr separation from nuclear reprocessing plants is based on several criteria: (1) Preferential adsorption of Xe and CO_2_ over Kr so these two gases can be separated in the first bed, and (2) Preferential adsorption of Kr over N_2_ and O_2_ so that Kr can be adsorbed in the second bed in more pure form with minimum waste volume. If the material fulfills these two criteria, then it should be qualified for the radiation stability. If the material does not achieve these two criteria, it is not helpful in this context to examine their radiation stability because they did not fulfill the main purpose of the Kr removal presented herein, which is the reduction of waste volume and Kr separation in more pure form with minimal amounts of other competing gases.

The single-component adsorption isotherms revealed that SIFSIX-3-Cu can preferentially adsorb CO_2_ and Xe over Kr, and Kr over N_2_ and O_2_; these findings have been supported by modeling. The practical use of SIFSIX-3-Cu in Kr capture and separation from nuclear fuel reprocessing off-gas was demonstrated by using a two-bed breakthrough technique at ambient conditions. SIFSIX-3-Cu successfully captures Xe in the first bed, while high purity Kr gas is captured in the second bed using the same material. We attribute the remarkable performance of SIFSIX-3-M family to the narrow pore size as well as the high polarizability of SiF_6_^−2^ anions. Indeed, modeling studies revealed that the adsorbate localizes between four neighboring SiF_6_^2–^ pillars within the small pores in this class of materials.

## Methods

### Synthesis

[M(pyz)_2_SiF_6_] (SIFSIX-3-M, M = Zn, Cu, Ni, Co, Fe) was synthesized by dissolving 10 mmol of pyrazine ligand (pyz) and 5 mmol of MSiF_6_ salt in 23 mL of methanol and heating the resulting solution at 75 °C for 3 days in a stainless steel Parr autoclave^8,29^. SIFSIX-3-Zn and SIFSIX-3-Co afford colorless and red crystals, respectively; while SIFSIX-3-Cu, SIFSIX-3-Ni, and SIFSIX-3-Fe afford blue, pale blue, and yellow crystalline powders, respectively. All structures possess six coordinated saturated metal centers that serve as 6-connected nodes with **pcu** topology through four equatorial pyrazine linkers and 2 axial SiF_6_^−2^ anion pillars to form the 3D-pillared square-grid nets.

### Gamma irradiation measurements

The MIT gamma irradiation facility, managed by the MIT Radiation Protection Program (RPP), houses a Gammacell 220 Excel self-shielded high dose-rate gamma ray irradiator (Supplementary Fig. 1) manufactured by MDS Nordion. The unit was manufactured in Canada by MDS Nordion on 13/10/2003 and contained an initial quantity of Cobalt-60 of 23,654 Curies (375.2 TBq) (Supplementary Fig. 2). The Co-60 sources are contained within a lead biological shield which allows for the safe use of the irradiator by trained radiation workers.

The Co-60 sources are arranged in a circle allowing for a uniform dose to the materials being irradiated. The samples ae loaded in the sample irradiation chamber and lowered by elevator to the Co-60 source array. The inside dimensions of the chamber are 6.10 in (15.49 cm) diameter and 8.06 in (20.47 cm) high. The current chamber dose rate is 4235 Rads/min (42.35 Gy/min). The original chamber dose rate was 32,228 Rads/min (322.28 Gy/min).

### Beta irradiation measurements

Experiments were performed at MIT’s High Voltage Research Laboratory (HVRL), which houses a continuous-wave Van de Graaff electron accelerator capable of producing electron kinetic energies of 1.5–3.0 MeV at beam currents of up to 30 μA. Samples were irradiated with 1.5 MeV electrons beam at a dose rate of 50 kGy/h.

### Powder X-ray diffraction measurements after gamma irradiation

The powder X-ray diffraction (PXRD) was collected on the PANalytical X’Pert Pro using 1.8 kW sealed X-ray tube source and Cu target. In order to solve the XRD data we use high score plus program to open the data and we used Rietveld and Pawley fitting methods.

## Supplementary information

Supplementary Information

Peer Review File

## Data Availability

All data needed to evaluate the conclusions of this paper are present in the paper and/or Supplementary Information. The source data underlying Figs. 3a–f, 4a, and 5a–c and Supplementary Figs. 3–7, 9, 10, 12–17, 19–22, and 30, CAR files that were used to make the different binding site pictures in Supplementary Figs. 23–29 and XYZ files containing the atomic coordinates corresponding to the Xe and Kr binding site pictures in Fig. 4b, c are provided as Source Data files. All source data are available from the corresponding author (S.K.E.) by request. Source data are provided with this paper.

## Source data

Source Data

## References

[1] Banerjee D (2015). Potential of metal–organic frameworks for separation of xenon and krypton. Acc. Chem. Res..

[2] Banerjee D, Simon CM, Elsaidi SK, Haranczyk M, Thallapally PK (2018). Xenon gas separation and storage using metal-organic frameworks. Chem.

[3] Riley BJ, Vienna JD, Strachan DM, McCloy JS, Jerden JL (2016). Materials and processes for the effective capture and immobilization of radioiodine: a review. J. Nucl. Mater..

[4] Zhang X (2017). Confinement of iodine molecules into triple-helical chains within robust metal–organic frameworks. J. Am. Chem. Soc..

[5] Soelberg NR (2013). Radioactive iodine and krypton control for nuclear fuel reprocessing facilities. Sci. Technol. Nucl. Ins..

[6] Banerjee, D. et al. Metal-organic framework with optimally selective xenon adsorption and separation. *Nat. Commun*. **7**, 11831 (2016). 10.1038/ncomms11831 PMC4909987 27291101

[7] Mohamed MH (2016). Hybrid ultra-microporous materials for selective xenon adsorption and separation. Angew. Chem. Int. Ed..

[8] Elsaidi SK (2017). Effect of ring rotation upon gas adsorption in SIFSIX-3-M (M = Fe, Ni) pillared square grid networks. Chem. Sci..

[9] Chen X (2015). Direct observation of Xe and Kr adsorption in a Xe-selective microporous metal–organic framework. J. Am. Chem. Soc..

[10] Wu T, Feng X, Elsaidi SK, Thallapally PK, Carreon MA (2017). Zeolitic imidazolate framework-8 (ZIF-8) membranes for Kr/Xe separation. Ind. Eng. Chem. Res..

[11] Hulvey Z (2013). Noble gas adsorption in copper trimesate, HKUST-1: an experimental and computational study. J. Phys. Chem. C..

[12] Perry JJ (2014). Noble gas adsorption in metal–organic frameworks containing open metal sites. J. Phys. Chem. C..

[13] Liu J, Strachan DM, Thallapally PK (2014). Enhanced noble gas adsorption in Ag@MOF-74Ni. Chem. Commun..

[14] Fernandez CA, Liu J, Thallapally PK, Strachan DM (2012). Switching Kr/Xe selectivity with temperature in a metal–organic framework. J. Am. Chem. Soc..

[15] Wang H (2014). The first example of commensurate adsorption of atomic gas in a MOF and effective separation of xenon from other noble gases. Chem. Sci..

[16] Bae Y-S (2013). High xenon/krypton selectivity in a metal-organic framework with small pores and strong adsorption sites. Micropor. Mesopor. Mat..

[17] Kancharlapalli S, Natarajan S, Ghanty TK (2019). Confinement-directed adsorption of noble gases (Xe/Kr) in MFM-300(M)-based metal–organic framework materials. J. Phys. Chem. C..

[18] Banerjee D, Elsaidi SK, Thallapally PK (2017). Xe adsorption and separation properties of a series of microporous metal–organic frameworks (MOFs) with V-shaped linkers. J. Mater. Chem. A.

[19] Liu J, Thallapally PK, Strachan D (2012). Metal–organic frameworks for removal of Xe and Kr from nuclear fuel reprocessing plants. Langmuir.

[20] Liu J (2014). A two-column method for the separation of Kr and Xe from process off-gases. Ind. Eng. Chem. Res..

[21] Forrest KA (2019). Investigating CO_2_ sorption in SIFSIX-3-M (M = Fe, Co, Ni, Cu, Zn) through computational studies. Cryst. Growth Des..

[22] Lee S-J (2016). Adsorptive separation of xenon/krypton mixtures using a zirconium-based metal-organic framework with high hydrothermal and radioactive stabilities. J. Hazard. Mater..

[23] Cadiau A (2017). Hydrolytically stable fluorinated metal-organic frameworks for energy-efficient dehydration. Science.

[24] Cui XL (2016). Pore chemistry and size control in hybrid porous materials for acetylene capture from ethylene. Science.

[25] Cadiau A, Adil K, Bhatt PM, Belmabkhout Y, Eddaoudi M (2016). A metal-organic framework-based splitter for separating propylene from propane. Science.

[26] Kanoo P (2012). Unusual room temperature CO_2_ uptake in a fluoro-functionalized MOF: insight from Raman spectroscopy and theoretical studies. Chem. Commun..

[27] Nugent P (2013). Porous materials with optimal adsorption thermodynamics and kinetics for CO_2_ separation. Nature.

[28] Uemura K, Maeda A, Maji TK, Kanoo P, Kita H (2009). Syntheses, crystal structures and adsorption properties of ultramicroporous coordination polymers constructed from hexafluorosilicate ions and pyrazine. Eur. J. Inorg. Chem..

[29] Elsaidi SK (2015). Hydrophobic pillared square grids for selective removal of CO_2_ from simulated flue gas. Chem. Commun..

[30] Shekhah O (2014). Made-to-order metal-organic frameworks for trace carbon dioxide removal and air capture. Nat. Commun..

[31] Yamamoto T, Ootsuka N (1982). Radiation damage of fluorocarbon by krypton-85 beta-rays, (4). J. Nucl. Sci. Technol..

[32] Chen L (2014). Separation of rare gases and chiral molecules by selective binding in porous organic cages. Nat. Mat..

[33] Allen F (2002). The Cambridge Structural Database: a quarter of a million crystal structures and rising. Acta Cryst. B.

[34] Desveaux BE (2019). CO_2_ behavior in a highly selective ultramicroporous framework: insights from single-crystal X-ray diffraction and solid-state nuclear magnetic resonance spectroscopy. J. Phys. Chem. C..

[35] Winger K, Feichter J, Kalinowski MB, Sartorius H, Schlosser C (2005). A new compilation of the atmospheric 85krypton inventories from 1945 to 2000 and its evaluation in a global transport model. J. Environ. Radioact..

[36] Moormann R, Kemp RS, Li J (2018). Caution is needed in operating and managing the waste of new pebble-bed nuclear reactors. Joule.

[37] Banerjee D (2016). Metal–organic framework with optimally selective xenon adsorption and separation. Nat. Commun..

[38] Thallapally PK, Grate JW, Motkuri RK (2012). Facile xenon capture and release at room temperature using a metal–organic framework: a comparison with activated charcoal. Chem. Commun..

[39] Munakata K, Yamatsuki S, Tanaka K, Fukumatsu T (2000). Screening test of adsorbents for recovery of krypton. J. Nucl. Sci. Technol..

