# Supplementary material for: Radiation-resistant metal-organic framework enables efficient separation of krypton fission gas from spent nuclear fuel
Source: Nat Commun. 2020 Jun 18;11:3103. doi: 10.1038/s41467-020-16647-1 (PMC7303119; doi:10.1038/s41467-020-16647-1)
Supplement: Supplementary file 1 — Supplementary Information [file 41467_2020_16647_MOESM1_ESM.pdf]

Supplementary Information

Radiation-resistant metal-organic framework enables efficient separation of krypton fission gas from spent nuclear fuel

Elsaidi et al.

Supplementary Note 1. Gamma Irradiation Study

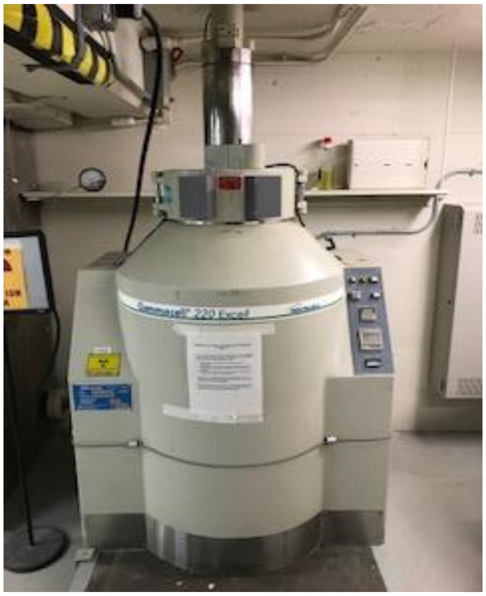

Supplementary Figure 1. Gammacell 220 Excel Irradiator.

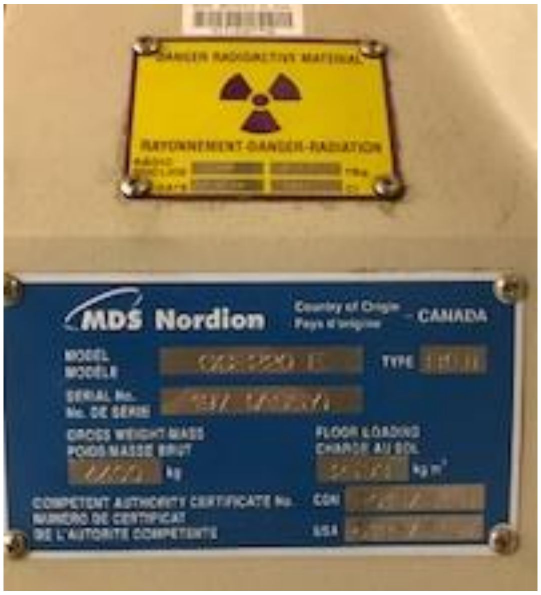

Supplementary Figure 2. Original Activity and Weight Specifications.

Supplementary Note 2. Powder X-ray diffraction after gamma irradiation

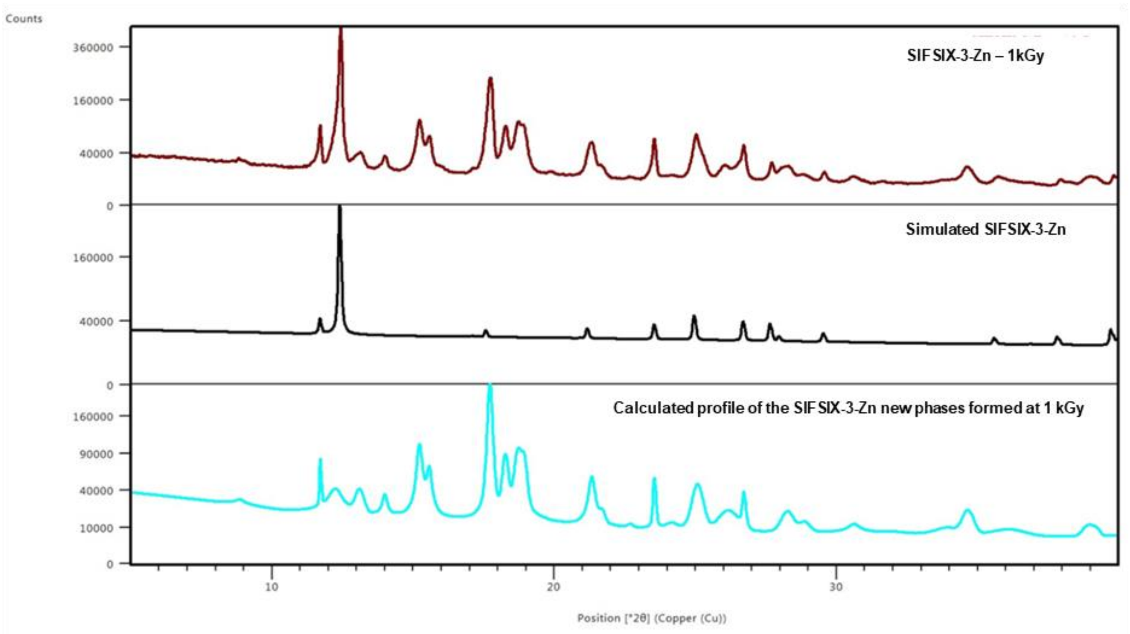

Supplementary Figure 3. 2D view comparison between SIFSIX-3-Zn after irradiation with 1 kGy (marron), the crystal structure of SIFSIX-3-Zn (black) and the calculated profile of the new phases (light blue).

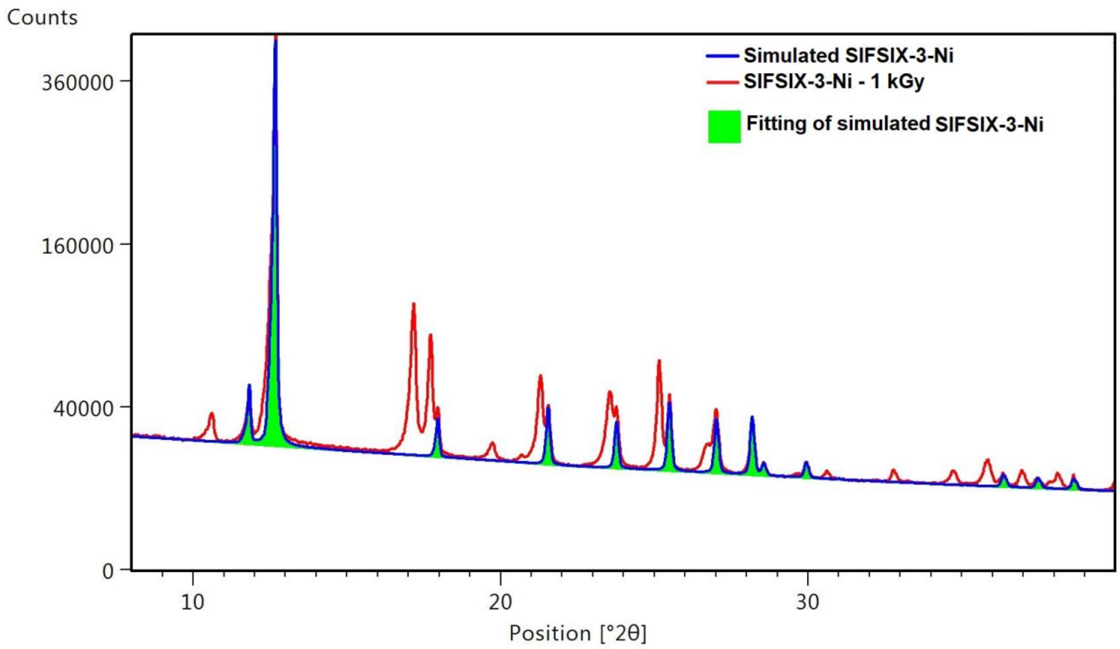

Supplementary Figure 4. Pawley fitting method between SIFSIX-3-Ni crystal structure (blue) and irradiated one at 1 kGy (red).

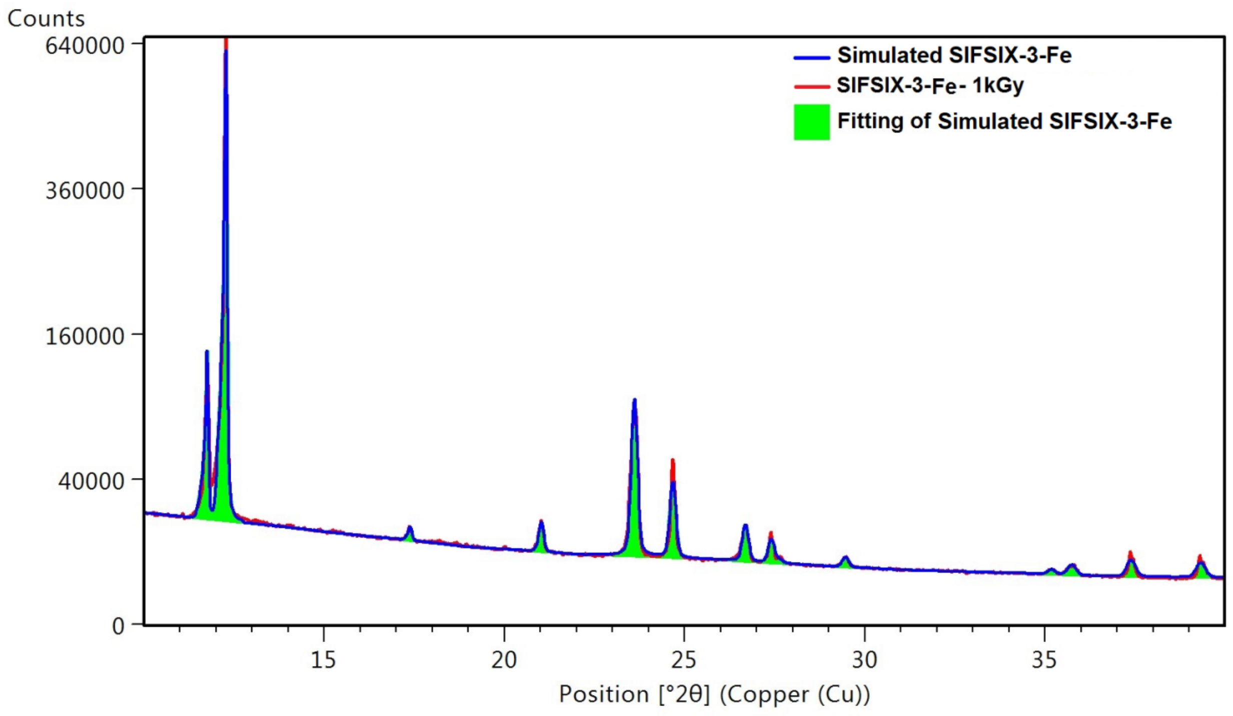

Supplementary Figure 5. Rietveld fitting method between SIFSIX-3-Fe crystal structure (blue) and irradiated structure at 1 kGy (red).

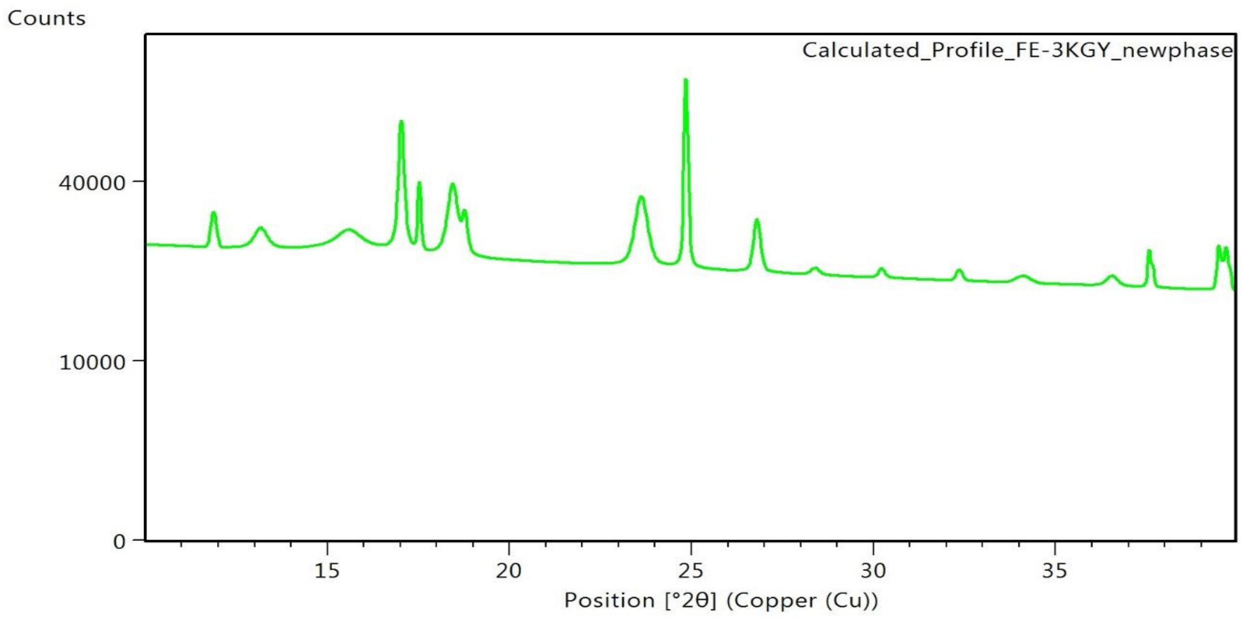

Supplementary Figure 6. Calculated profile for the new phase appears after irradiation of SIFISIX-3-Fe with 3 kGy.

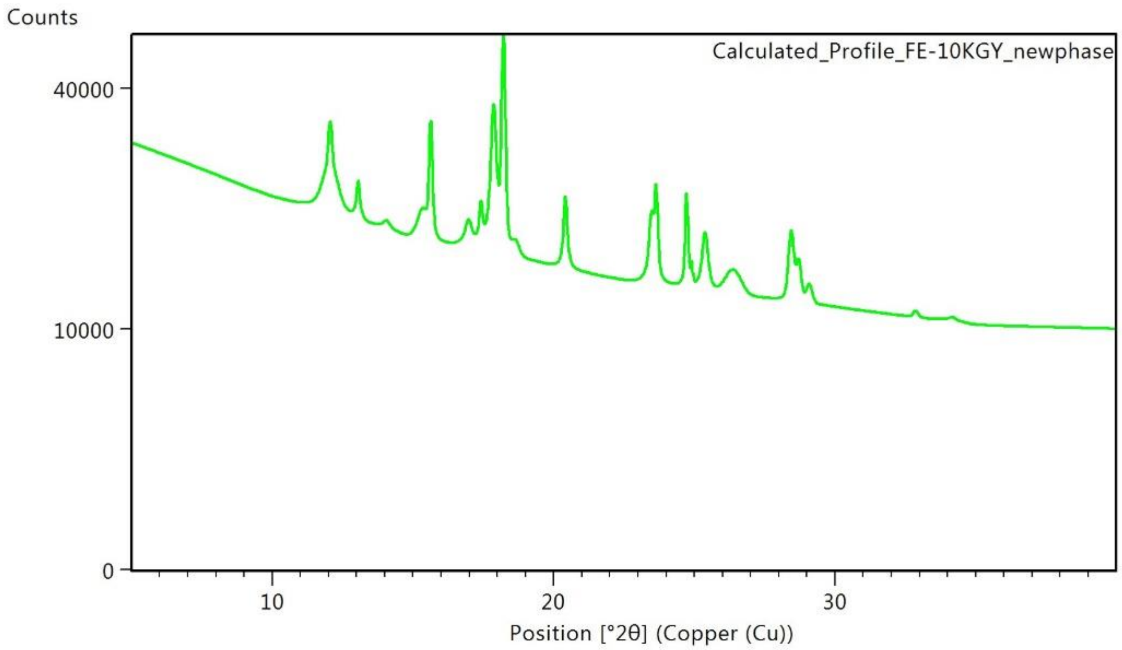

Supplementary Figure 7. Calculated profile for the new phase appears after irradiation of SIFISIX-3-Fe with 10 kGy.

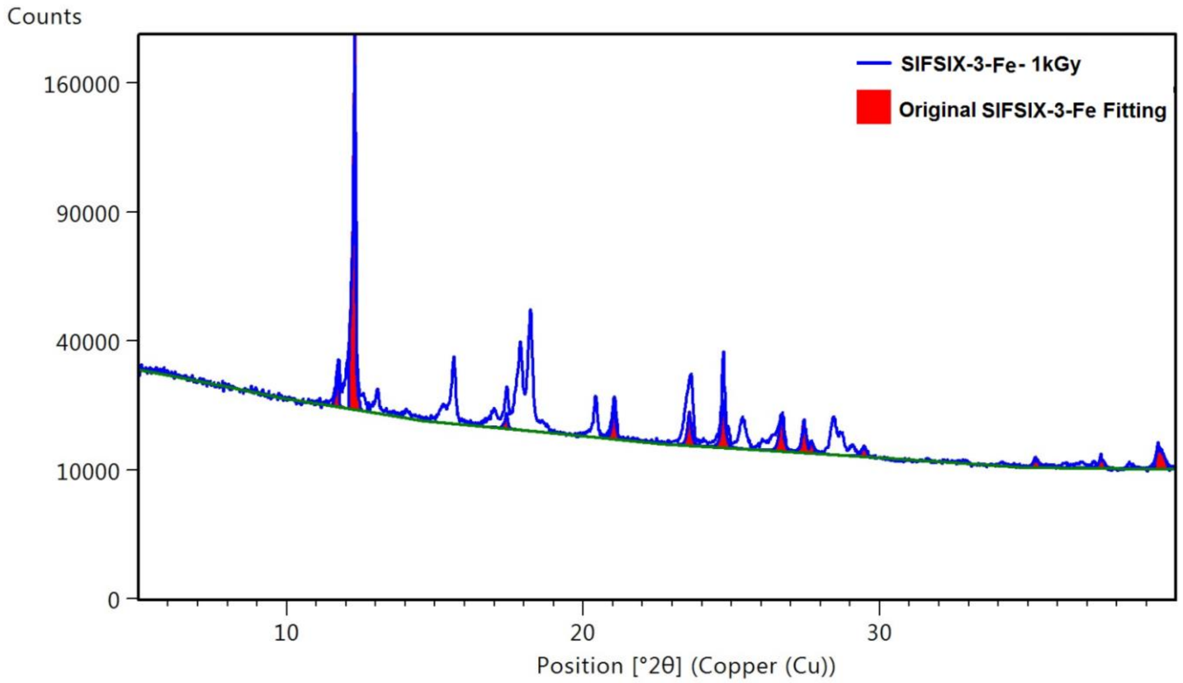

Supplementary Figure 8. Pawley fitting method between SIFSIX-3-Fe crystal structure and irradiated one at 10 kGy (green color for the baseline).

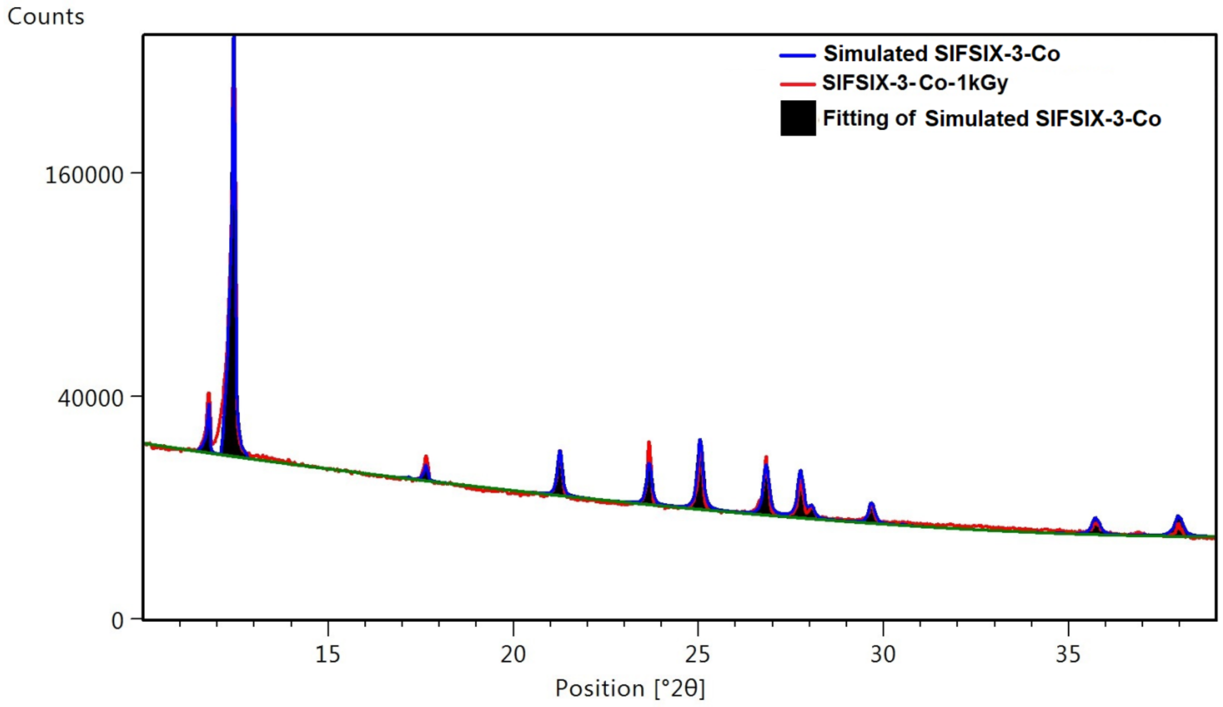

Supplementary Figure 9. Rietveld fitting method between SIFSIX-3-Co crystal structure (blue) and irradiated structure at 1 kGy (red). Baseline is shown in green.

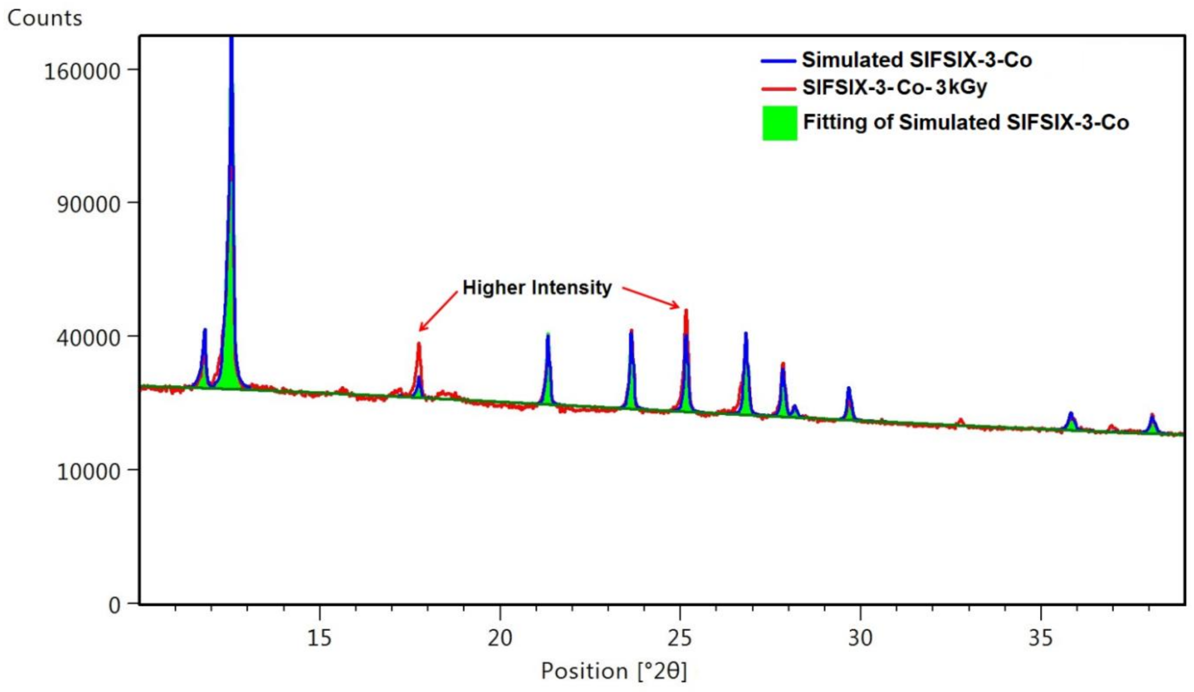

Supplementary Figure 10. Rietveld fitting method between SIFSIX-3-Co crystal structure (blue) and irradiated structure at 3 kGy (red).

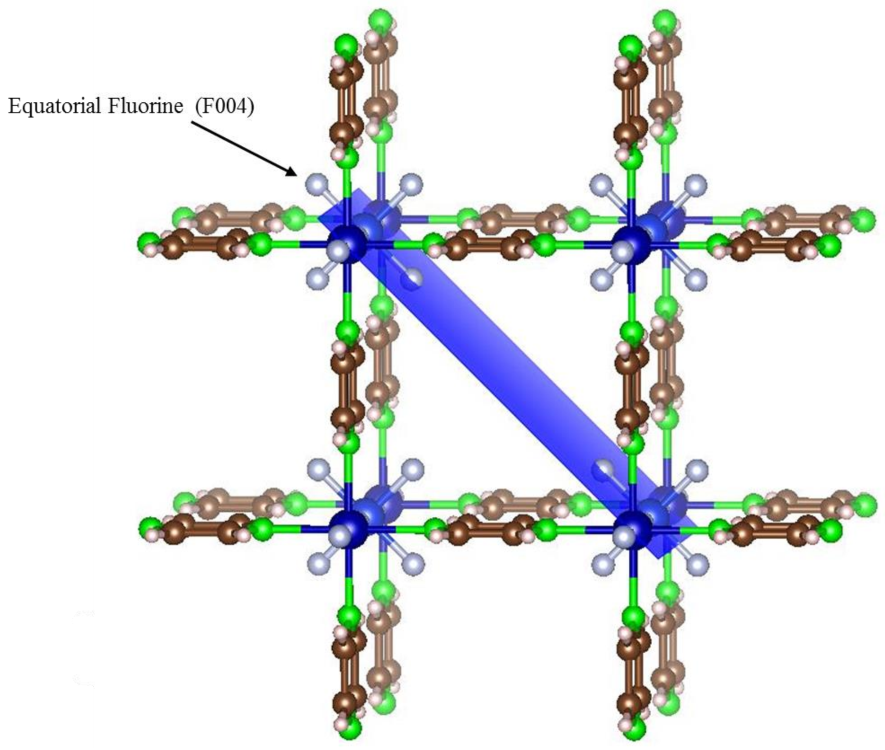

Supplementary Figure 11. Crystal structure of SIFSIX-3-Co -110 plane.

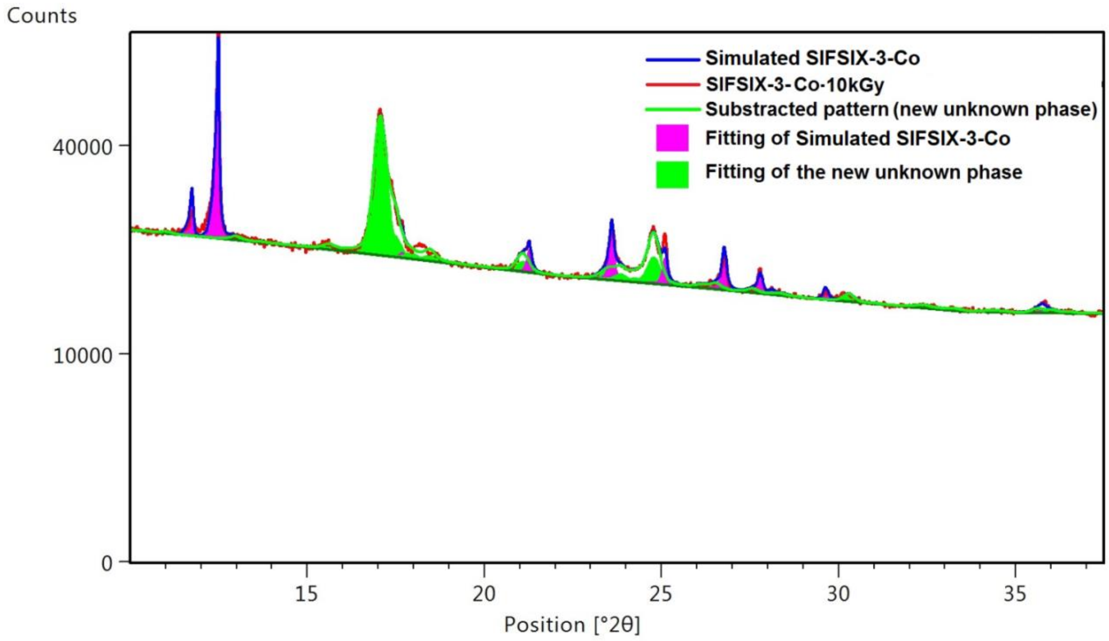

Supplementary Figure 12. Pawley fitting method between SIFSIX-3-Co crystal structure (blue) and irradiated structure at 10 kGy (red). To quantitatively analyze the two superimposed XRD patterns we apply both Rietveld and Pawley fitting methods to the SIFSIX-3-Co and the unknown phase, respectively. The Pawley method is used to obtain structure factors by fitting the peak intensities independently.

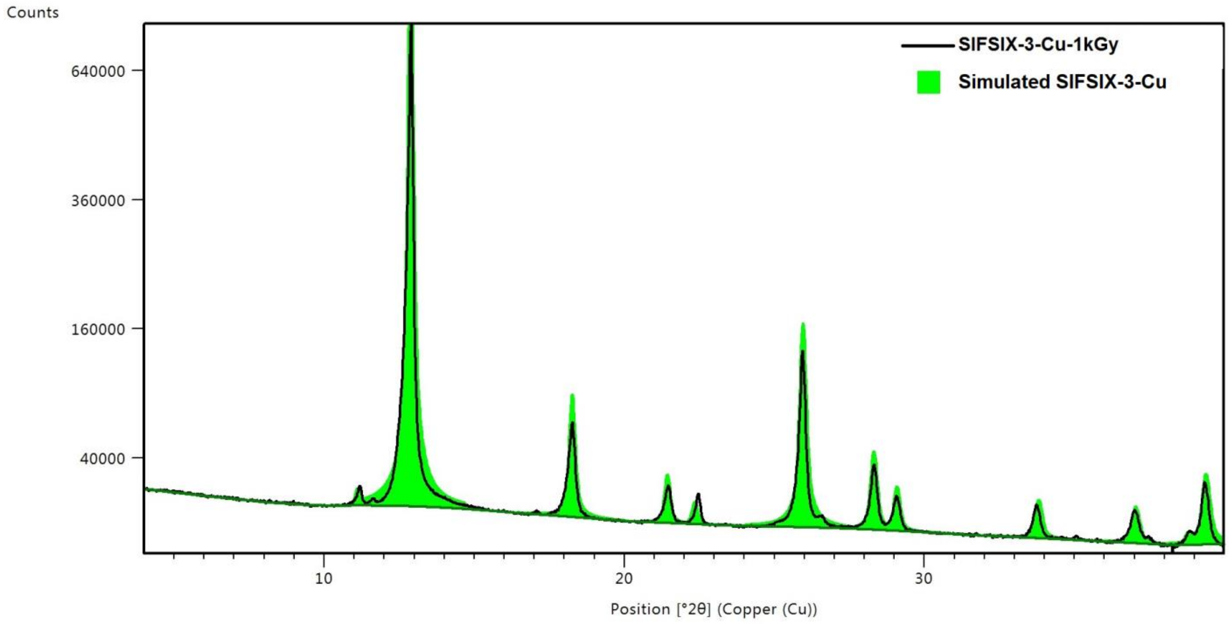

Supplementary Figure 13. Pawley fitting method between SIFSIX-3-Cu crystal structure and irradiated one at 1 kGy.

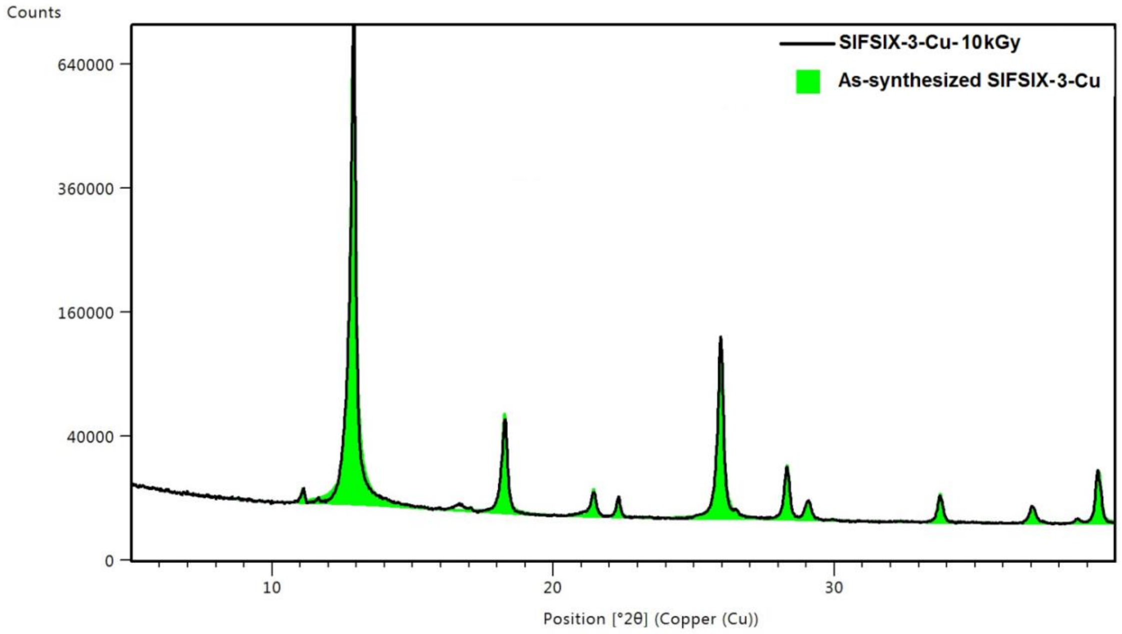

Supplementary Figure 14. Pawley fitting method between SIFSIX-3-Cu crystal structure and irradiated one at 10 kGy.

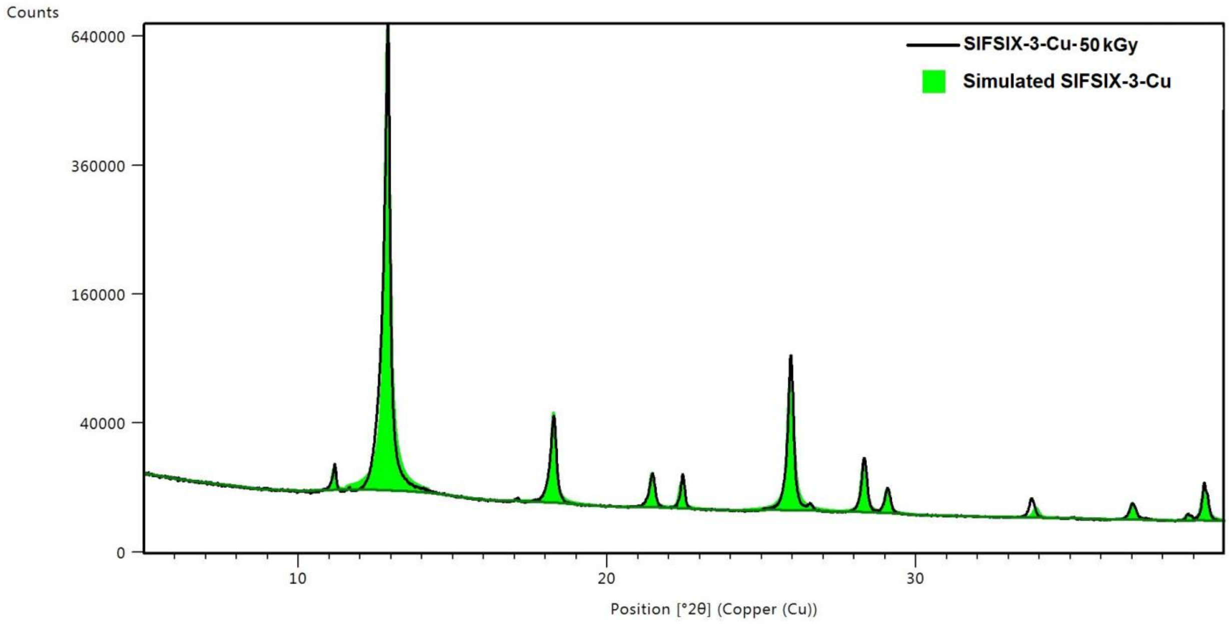

Supplementary Figure 15. Pawley fitting method between SIFSIX-3-Cu crystal structure and irradiated one at 50 kGy.

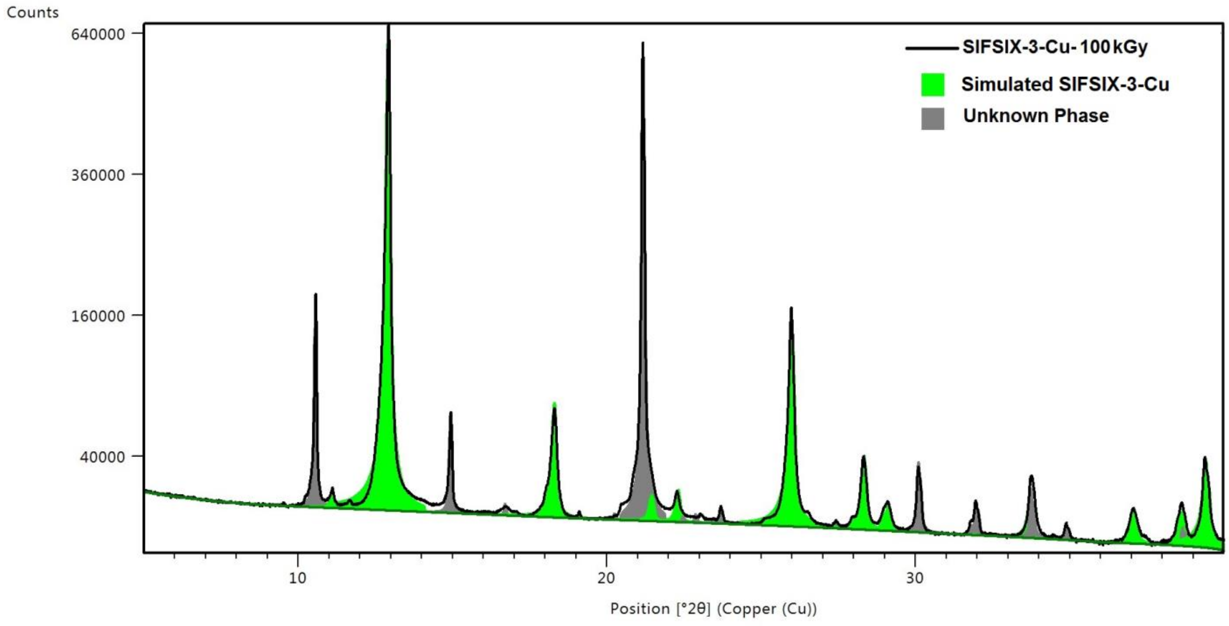

Supplementary Figure 16. Pawley fitting method between SIFSIX-3-Cu crystal structure and irradiated one at 100 kGy.

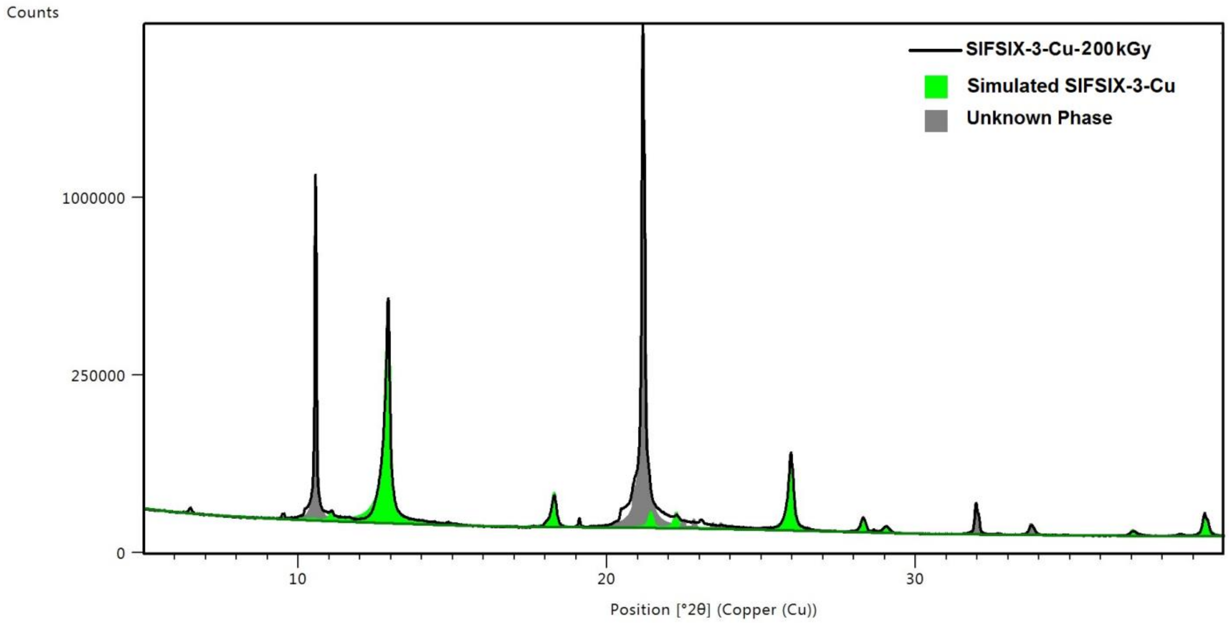

Supplementary Figure 17. Pawley fitting method between SIFSIX-3-Cu crystal structure and irradiated one at 200 kGy.

Supplementary Note 3. Beta Irradiation Study

Beta Dose Calculations from Spent Nuclear Fuel to the SIFSIX-3-Cu MOF:

^{85}Kr concentration is reported to be 130 to 1800 TBq/Mg (3.51E3 to 4.86E4 Ci/Mg) of spent fuel.[1] Calculations of absorbed dose from ^{85}Kr processed in an adsorption matrix is necessary to compare to the damage threshold to that matrix.

^{85}Kr's maximum beta energy for the predominate emission (99.57%) is 687 keV and has a corresponding average beta energy of 251 keV. The energy spatial equilibrium dose rate is represented by the total amount of energy deposited per gram of matrix (SIFSIX-3-Cu).[2]

$$\dot{D} = \frac{A}{m} * 3.7E10 * Y * \bar{T}_\beta * 3600\frac{s}{h} * 1.602 * 10^{-10}\frac{Gy}{Mev/g}$$

Where: A is the Activity in Ci,

m is the mass of the matrix volume (assume 1 gm of MOF),

Y is the yield for the transition of interest (assume 1 for beta particles),

T_β is the average kinetic energy of the beta particle (0.251 MeV), and

All others are conversion factors:

3.7E10 dps per curie,

3600 sec per hour, and

$1.602 * 10^{-10}\frac{Gy}{Mev/g}$

Simplifying the equation, the dose rate range in Gy/hr in one gram of SIFSIX-3-Cu is as follows:

$$\dot{D} = 5.34E3 * \frac{A}{m}\frac{Gy}{h}$$

Thus, the dose rate range for the Kr-85 activity is:

$$\dot{D}(130\,TBq) = 1.87E4\frac{KGy}{hr}$$

$$\dot{D}(1800\,TBq) = 2.64\text{E}5\frac{KGy}{hr}$$

Therefore, the radiation dose rate to 1 gm SIFSIX-3-Cu from 1 gm of spent nuclear fuel:

$\dot{D}$ = 1.87E-2 kGy/h (130 TBq/Mg) or

$\dot{D}$ = 2.648E-1 kGy/h (1800 TBq/Mg)

According to the obtained results from both beta and gamma irradiation experiments, SIFSIX-3-Cu is radiation resistant up to 50 KGy. Hence, 1 gm of SIFSIX-3-Cu can separate ^{85}Kr effectively from 2673.79 g spent nuclear fuel (130 TBq/Mg case) or 188 g spent nuclear fuel (1800 TBq/Mg case) without any crystal structure damage, if keeping all the ^{85}Kr inside for 1 hour.

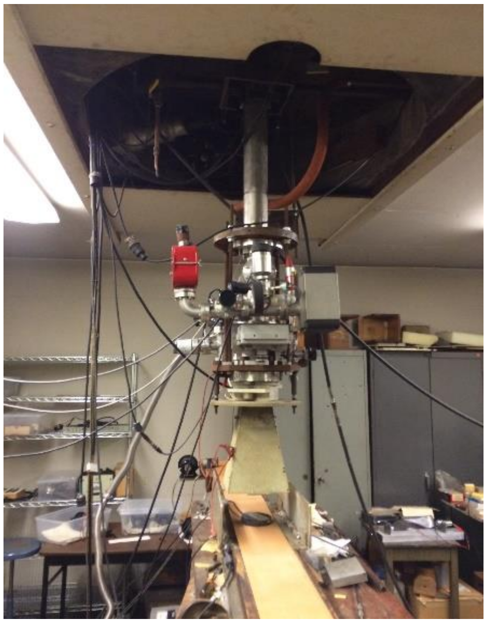

Supplementary Figure 18. Van deGraaff electron accelerator.

Supplementary Note 4. Gas adsorption studies

The single-component gas adsorption isotherms were collected on Quantachrome Q1 surface area and gas analyzer instrument within the P/P_o range of 0–1.0.

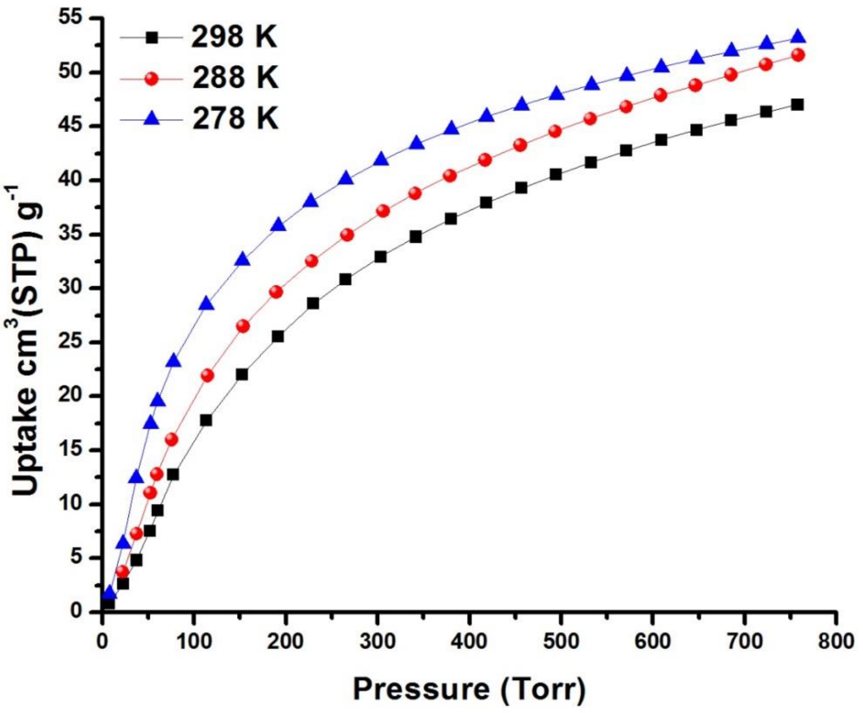

Supplementary Figure 19. Single component Xe sorption isotherms for SIFSIX-3-Cu measured at different temperatures.

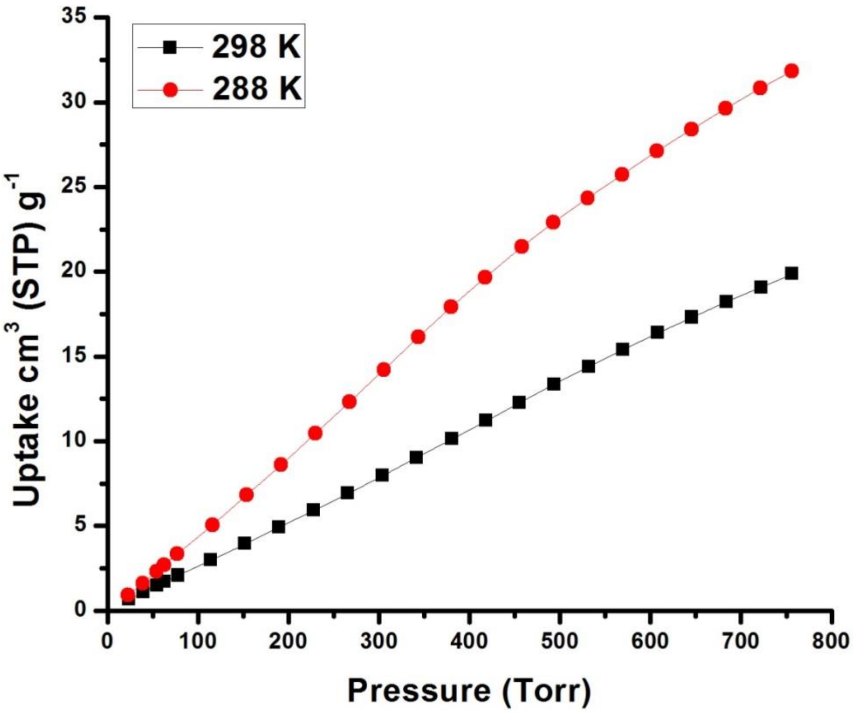

Supplementary Figure 20. Single component Kr sorption isotherms for SIFSIX-3-Cu measured at 298 K and 288 K.

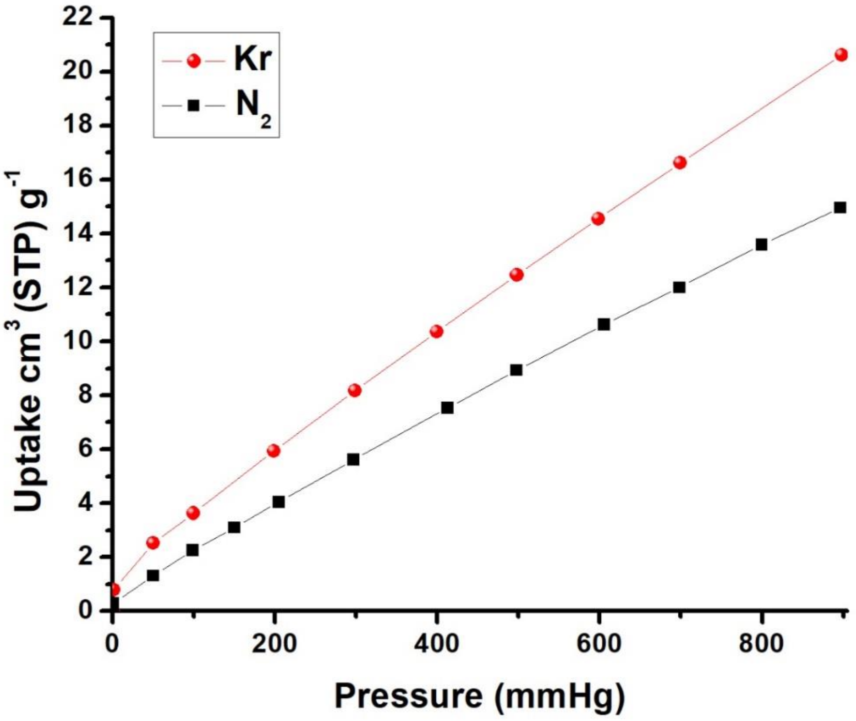

Supplementary Figure 21. Single component Kr and N_2 sorption isotherms for Ni-MOF-74 measured at 298 K demonstrating the low Kr/N_2 selectivity.

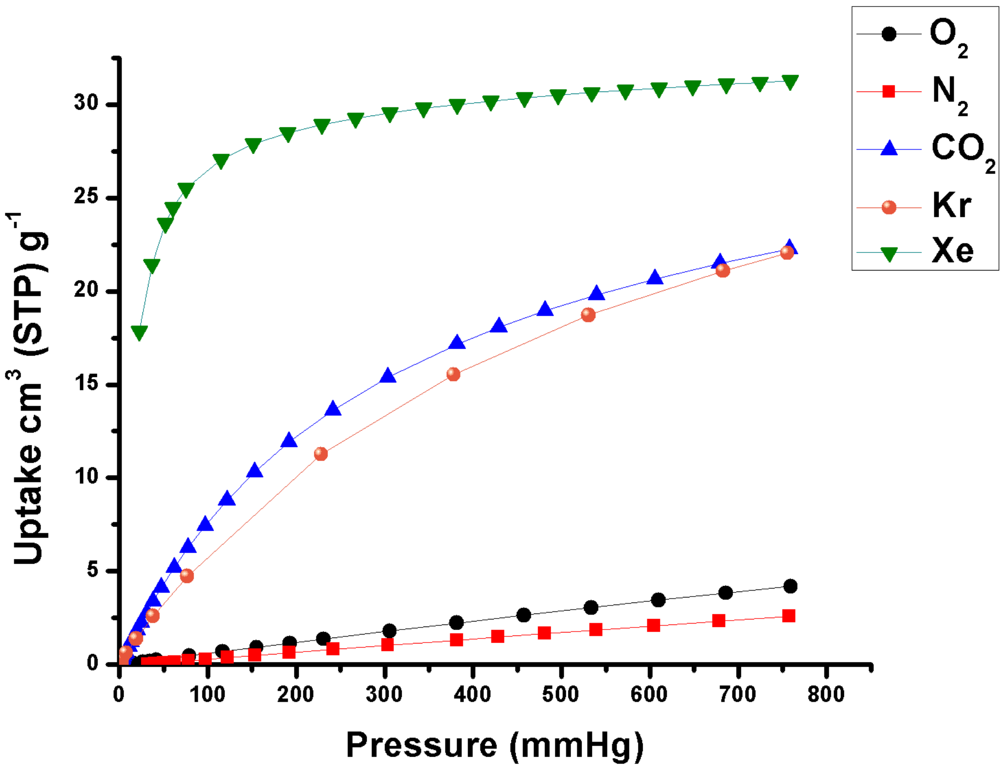

Supplementary Figure 22. Single component Xe, CO_2, Kr, O_2 and N_2 sorption isotherms for SBMOF-1 measured at 298 K demonstrating extremely low Kr/CO_2 selectivity.

Supplementary Note 5. Modeling Studies in SIFSIX-3-Cu

Since SIFSIX-3-Cu was found to be the most stable analogue when subjected to gamma radiation, it was the focus of molecular simulation studies of Xe and Kr adsorption. To the best of our knowledge, theoretical studies of Xe and Kr adsorption in the Cu variant of SIFSIX-3-M have not been performed earlier.

First, the originally published crystal structure of SIFSIX-3-Cu (as taken from reference 3) was fully optimized (i.e., atoms and lattice parameters relaxed) using the Vienna *ab initio* Simulation Package (VASP)[4] (version 5.4.4) with the projector augmented wave (PAW) method[5], Perdew–Burke–Ernzerhof (PBE) functional,[6] and the DFT-D2 correction method of Grimme.[7] This optimized structure was then used to carry out classical simulated annealing calculations[8] of Xe and Kr in the material to determine the most favorable binding site for the respective adsorbates. These simulations were performed within the 3 × 3 × 3 supercell of the material through a Monte Carlo process. We note that the crystal structure of SIFSIX-3-Cu was also optimized using the DFT-D3 method of Grimme *et al*.[9] However, the resulting relaxed structure had an energy that was surprisingly higher than that obtained using the DFT-D2 method (–705.5814605 vs –706.2898731 eV). Since the DFT-D2 method generated a crystal structure that was more stable, this optimized structure was therefore utilized for the classical simulations.

The classical force field for SIFSIX-3-Cu, which includes Lennard-Jones 12–6 parameters, point partial charges, and scalar point polarizabilities, was established in previous work[10] and used for the simulations executed herein. Simulated annealing calculations within the canonical (*NVT*) ensemble were implemented for a single Xe and Kr atom, respectively, in SIFSIX-3-Cu using polarizable potentials that were developed previously for the individual adsorbates.[11] These simulations started at an initial temperature of 500 K, and this temperature was scaled by a factor of 0.99999 after every 1,000 Monte Carlo steps. The simulations continued until the temperature of the MOF–adsorbate system decreased below 10 K. These calculations were performed using the Massively Parallel Monte Carlo (MPMC) code.[12]

The global minimum for both Xe and Kr in SIFSIX-3-Cu was identified as localization between the equatorial fluorine atoms of four neighboring SiF_6^{2-} anions within the square corridor (Supplementary Figs 23 and 24). The greater atomic radius for Xe relative to Kr (1.08 vs 0.88 Å) afforded a better fit within the pores and generally shorter distances between the adsorbate and the equatorial F atoms (r = 0.42 Å), which results in stronger interactions with the host. After subtracting the distance corresponding to the atomic radii of the adsorbate and F atoms from the distance measured between the two center-of-masses, the Xe⋯F distances for the annealed position were measured to be 1.94, 1.96, 1.98, and 2.00 Å, while distances of 2.14, 2.18, 2.22, and 2.25 Å were measured for the Kr⋯F interaction.

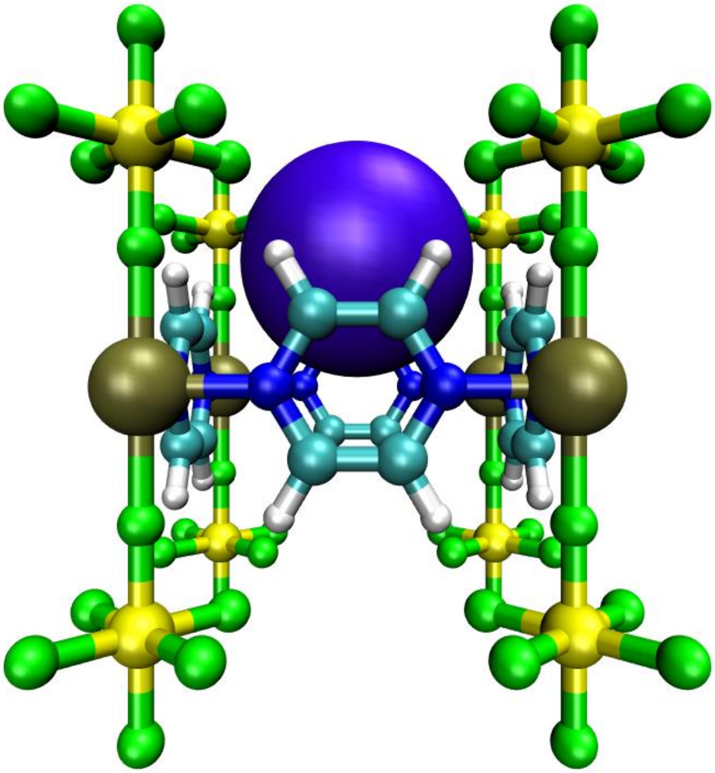

Supplementary Figure 23. Molecular illustration of the most favorable binding site for Xe in **SIFSIX-3-Cu** (side view) as determined from simulated annealing calculations. Atom colors: C = cyan, H = white, N = blue, F, = green, Si = yellow, Cu = gold, Xe = violet.

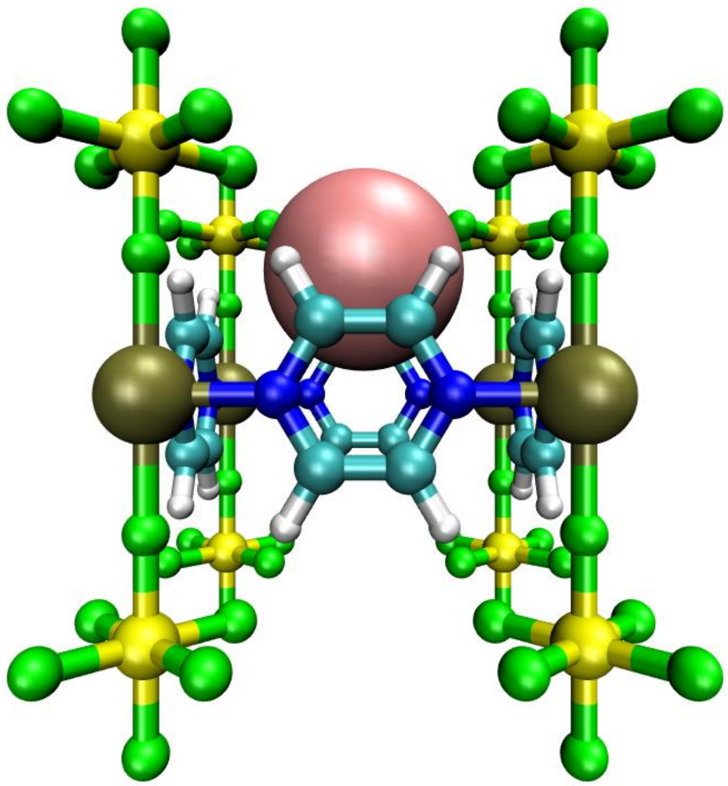

Supplementary Figure 24. Molecular illustration of the most favorable binding site for Kr in **SIFSIX-3-Cu** (side view) as determined from simulated annealing calculations. Atom colors: C = cyan, H = white, N = blue, F, = green, Si = yellow, Cu = gold, Kr = pink.

Periodic DFT calculations were also performed to evaluate the adsorption energy (Δ*E*) for Xe, Kr, CO_2, N_2, and O_2 within SIFSIX-3-Cu. These calculations were performed using VASP with the same methods that were employed for optimizing an empty unit cell of the material. The position of a single atom/molecule of each adsorbate was initially optimized within the rigid unit cell of the MOF. Afterward, another optimization was carried out in which the position of all atoms and lattice parameters of the system could fluctuate. The optimized position of a Xe, Kr, CO_2, N_2, and O_2 atom/molecule about four neighboring SiF_6^{2-} anions within SIFSIX-3-Cu are displayed in Supplementary Figs 25-29. The DFT-optimized positions for Xe and Kr in this material are visually identical to those obtained through simulated annealing calculations (Supplementary Figs 23-24).

The Δ*E* for the adsorbates localized within the unit cell of SIFSIX-3-Cu were calculated by the following:

$$\Delta E = E(\text{MOF + Adsorbate}) - E(\text{MOF}) - E(\text{Adsorbate})$$

where E(MOF + Adsorbate) is the energy of the unit cell of the MOF with the adsorbate, E(MOF) is the energy of the empty unit cell, and E(Adsorbate) is the energy of the adsorbate. The calculated ΔE values for Xe, Kr, CO_2, N_2, and O_2 within SIFSIX-3-Cu are listed in Supplementary Table 1.

Supplementary Table 1: Calculated adsorption energies (in kJ mol^{-1}) for a single atom/molecule of Xe, Kr, CO_2, N_2, and O_2 within the unit cell of SIFSIX-3-Cu as determined from periodic DFT calculations using VASP.

**Absorbate**	**ΔE (kJ mol^{-1})**
Xe	–45.93
Kr	–31.14
CO_2	–58.42
N_2	–21.01
O_2	–15.10

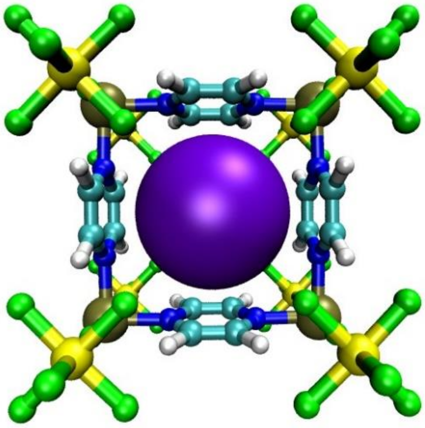

(a)

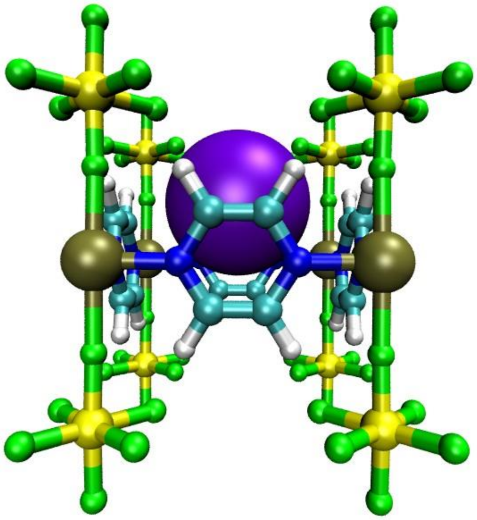

(b)

Supplementary Figure 25. Molecular illustration of the most favorable binding site for Xe in SIFSIX-3-Cu as determined from periodic DFT calculations using VASP: (a) top view; (b) side view. Atom colors: C = cyan, H = white, N = blue, F, = green, Si = yellow, Cu = gold, Xe = violet.

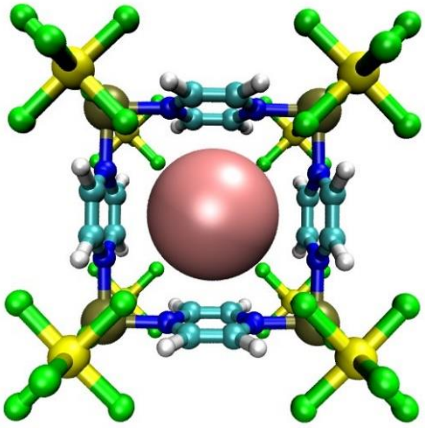

(a)

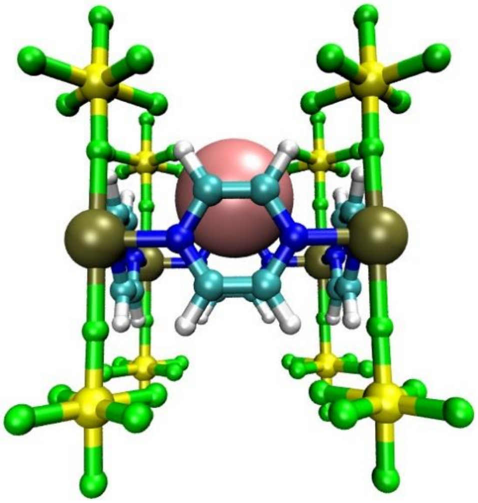

(b)

Supplementary Figure 26. Molecular illustration of the most favorable binding site for Kr in SIFSIX-3-Cu as determined from periodic DFT calculations using VASP: (a) top view; (b) side view. Atom colors: C = cyan, H = white, N = blue, F, = green, Si = yellow, Cu = gold, Kr = pink.

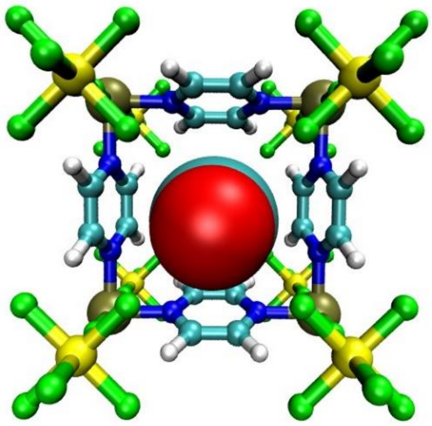

(a)

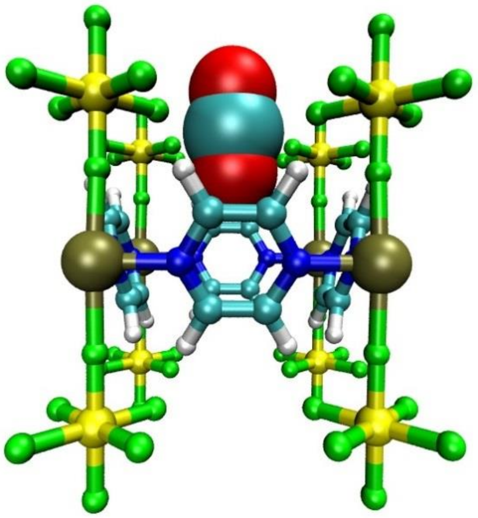

(b)

Supplementary Figure 27. Molecular illustration of the most favorable binding site for CO_2 in SIFSIX-3-Cu as determined from periodic DFT calculations using VASP: (a) top view; (b) side view. Atom colors: C = cyan, H = white, N = blue, O = red, F, = green, Si = yellow, Cu = gold.

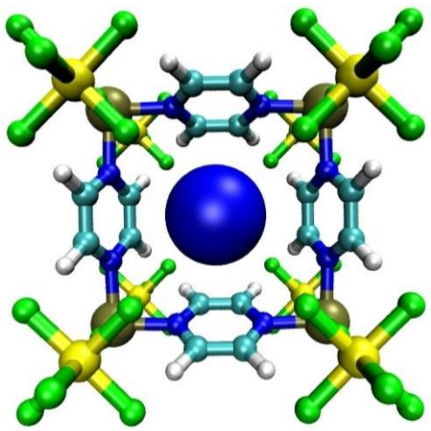

(a)

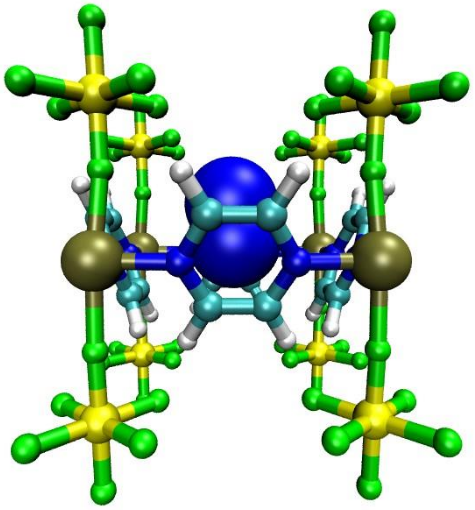

(b)

Supplementary Figure 28. Molecular illustration of the most favorable binding site for N_2 in SIFSIX-3-Cu as determined from periodic DFT calculations using VASP: (a) top view; (b) side view. Atom colors: C = cyan, H = white, N = blue, F, = green, Si = yellow, Cu = gold.

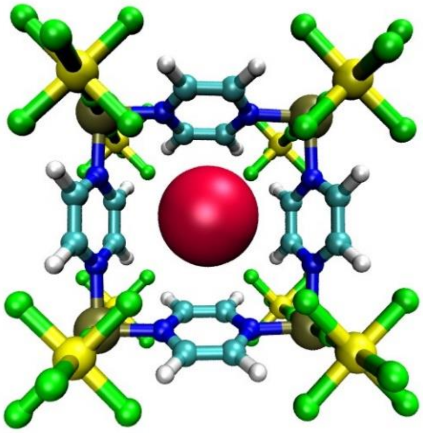

(a)

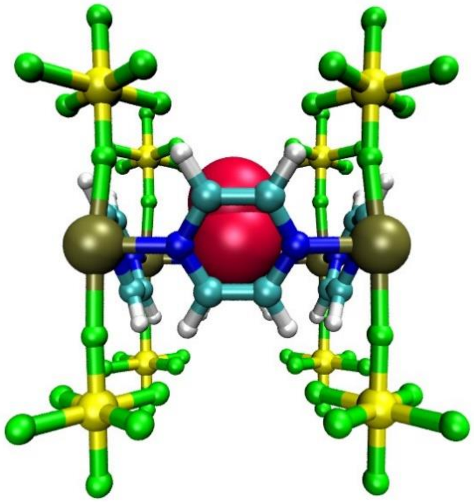

(b)

Supplementary Figure 29. Molecular illustration of the most favorable binding site for O_2 in SIFSIX-3-Cu as determined from periodic DFT calculations using VASP: (a) top view; (b) side view. Atom colors: C = cyan, H = white, N = blue, O = red, F, = green, Si = yellow, Cu = gold.

Supplementary Note 6. Single-bed breakthrough experiments:

Experimental single-bed breakthrough measurements were conducted by packing about 1 g of adsorbent sample in the column. SIFSIX-3-Cu was activated under vacuum at room temperature for 12 hr and at 55 °C for another 12 hr. Pressurization of the column-containing adsorbent material was accomplished by syringe pump (Teledyne ISCO) directly connected to the system. An inline pressure transducer was used to verify column pressure. The column was cooled to room temperature and the pure He gas was initially flowed to a Stanford Research Residual Gas Analyzer (RGA) for first three minutes, after which the flow of He is stopped and flow of the gas mixture is introduced to the fixed bed column containing the adsorbent sample with flow rate of 5 ml/min and total pressure of 1 bar at room temperature. Effluent gases were thereby tracked with the RGA, while the gases breaking through the column were indicated by an increase in the pressure. This ran for the next 2-4 hours. The experimental set-up of the single-bed breakthrough experiment is presented in Supplementary Scheme 1.

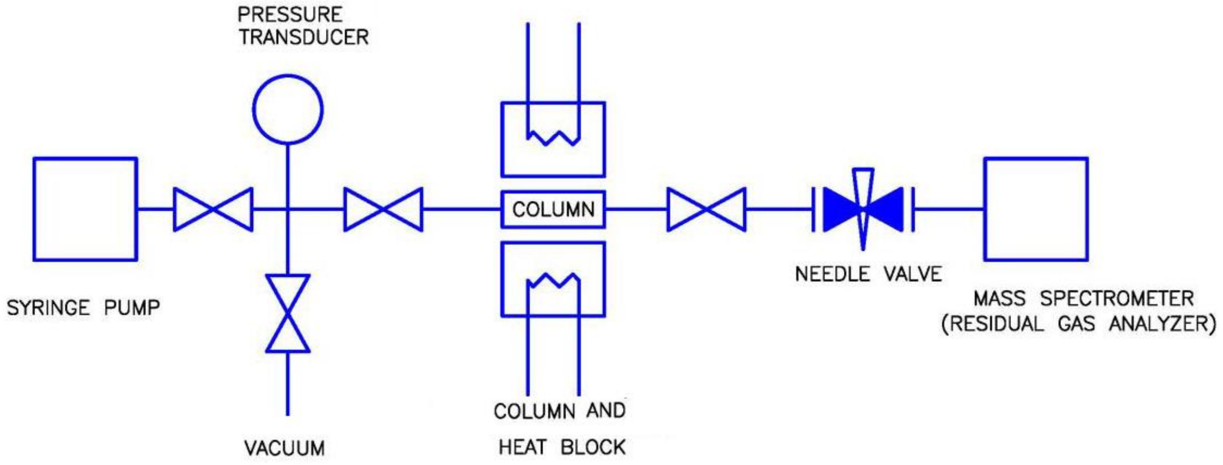

Supplementary Scheme 1. Schematic representation of the single column breakthrough experiment set-up combined with the mass spectrophotometer.

Supplementary Note 7. Two-bed breakthrough experiments for Xe/Kr gas mixture

The breakthrough apparatus used in the current experiments was previously described in literature.[13] It consists of two adsorption beds in series equipped with two separate temperature controllers and one common mass spectrometer. Mass flow controllers are used to control the gas flow through the columns by adjusting the valve in order to switch the system between one-bed or two-bed regimes. Both columns have the same diameter of 10 mm and length of 100 mm and the voidage of the packed bed e equals to 0.5. The pressurization of the two beds containing the SIFSIX-3-Cu was implemented by syringe pump (Teledyne ISCO) directly connected to the system. An inline pressure transducer was used to verify column pressure. At the other side, Stanford Research Residual Gas Analyzer (RGA) was connected to the system in order to track the effluent gases, while the gases breaking through the two-columns were indicated by an increase in the pressure. The simulated nuclear gas-mixture is composed of 400 ppm Xe, 40 ppm Kr, balanced with air. The gas-flow can be switched between one-bed mode and two bed mode by adjusting the valve in between the beds. Prior to the breakthrough experiments, adsorbent sample was activated at appropriate temperature under He flow and the total flow rate was kept constant until the start of the breakthrough experiment. The inlet and outlet pressure was monitored by pressure gauge and kept constant at 1 bar. All flow rate for both the gas-mixture and He flow was 5 ml/min. Prior to the two-bed experiment, gas-mixture was flown through bed one only to evaluate the Xe adsorption capacity under dynamic condition and current setup. For the two-bed experiment, once the Kr (and other gaseous components) breaks through the bed and reaches equilibrium concentration, the resultant gas-flow was switched to the 2nd bed using the valve in between the bed. Once Kr and other gaseous component breakthrough from the 2nd bed as recorded in the mass spectrometer, the valve to the 2nd bed was turned off, so that gas-mix breaking through the 1st bed only reaches the mass–spectrometer. The feed gas tank (400 ppm Xe, 40 ppm Kr, balance air, also termed as simulated off-gas stream) were purchased from Advanced Specialty Gases (Reno, NV).

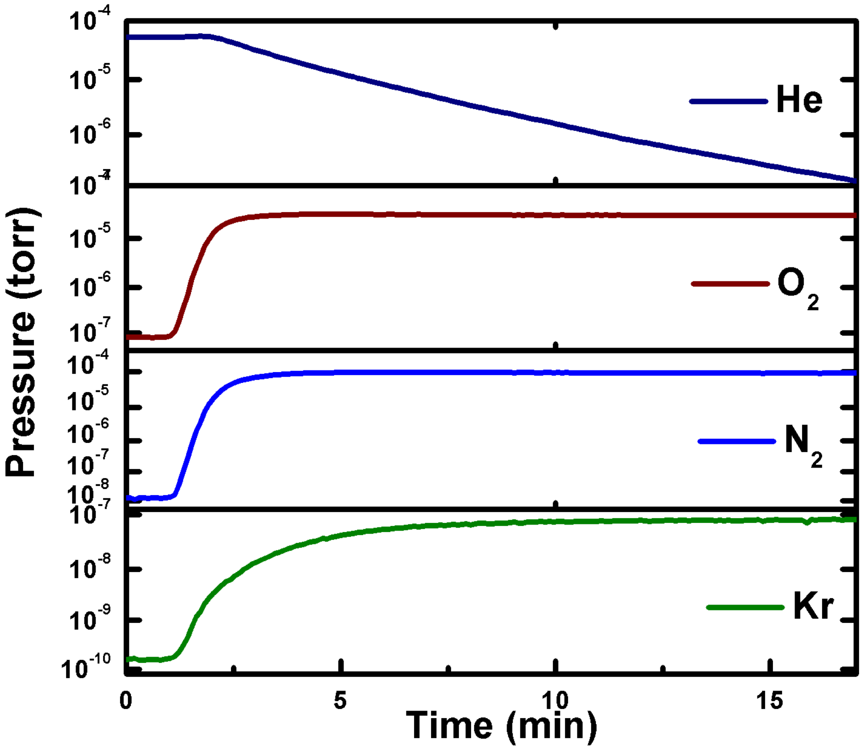

Supplementary Figure 30. Single-bed breakthrough experiment using 1000 ppm Kr balanced with dry air for Ag mordenite demonstrating the low Kr/N_2 selectivity.

Supplementary References

1. Wingera, K.; Feichtera, J.; Kalinowskib, M.B.; Sartoriusc, H.; Schlosser. C. *Journal of Environmental Radioactivity*, **2005**, *80*, 183–215.
2. Attix, F., H. **2004** *WILEY-VCH Verlag GmbH & Co. KGaA.* ISBN:9780471011460.
3. Shekhah, O.; Belmabkhout, Y.; Chen, Z.; Guillerm, V.; Cairns, A.; Adil, K.; Eddaoudi, M. *Nat. Commun*. **2014**, *5*, 4228.
4. (a) Kresse, G.; Hafner, J. *Phys. Rev. B* **1993**, *47*, 558–561. (b) Kresse, G.; Hafner, J. *Phys. Rev. B* **1994**, *49*, 14251–14269. (c) Kresse, G.; Furthmüller, J. *Comput. Mater. Sci*. **1996**, *6*, 15–50. (d) Kresse, G.; Furthmüller, J. *Phys. Rev. B*. **1996**, *54*, 11169–11186.
5. (a) Blöchl, P. E. *Phys. Rev. B* **1994**, *50*, 17953. (b) Kresse, G.; Joubert, D. *Phys. Rev. B* **1999**, *59*, 1758.
6. (a) Perdew, J. P.; Burke, K.; Ernzerhof, M. *Phys. Rev. Lett*. **1996**, *77*, 3865. (b) Perdew, J. P.; Burke, K.; Ernzerhof, M. *Phys. Rev. Lett*. **1997**, *77*, 1396.
7. Grimme, S. *J. Comput. Chem*. **2006**, *27*, 1787–1799.
8. Grimme, S.; Antony, S.; Ehrlich, S.; Krieg, H. *J. Comput. Chem*. **2010**, *132*, 154104.
9. Kirkpatrick, S.; Gelatt, C. D.; Vecchi, M. P. *Science* **1983**, 220, 671−680.
10. Forrest, K. A.; Pham, T.; Space, B. *CrystEngComm* **2017**, *19*, 3338–3347.
11. Mohamed, M. H.; Elsaidi, S. K.; Pham, T.; Forrest, K. A.; Schaef, H. T.; Hogan, A.; Wojtas, L.; Xu, W.; Space, B.; Zaworotko, M. J.; Thallapally, P. K. *Angew. Chem. Int. Ed.* **2016**, *55*, 8285–8289.
12. Belof, J. L.; Space, B. *Massively Parallel Monte Carlo (MPMC)*. 2012, Available on GitHub. https://github.com/mpmccode/mpmc.
13. Liu, J.; Fernandez, C. A.; Martin, P. F.; Thallapally, P. K.; Strachan, D. M. *Industrial & Engineering Chemistry Research* **2014**, *53*, 12893.
