# Supplementary material for: Radiation-resistant metal-organic framework enables efficient separation of krypton fission gas from spent nuclear fuel
Source: Nat Commun. 2020 Jun 18;11:3103. doi: 10.1038/s41467-020-16647-1 (PMC7303119; doi:10.1038/s41467-020-16647-1)
Supplement: Supplementary file 2 — Peer Review File [file 41467_2020_16647_MOESM2_ESM.pdf]

Reviewers' comments:

Reviewer #1 (Remarks to the Author):

The authors found that among the series of SIFSIX-3 MOFs, SIFSIX-3-Cu is good for 85Kr removal from nuclear reprocessing plants because it is radioactively stable and can selectively separate and store 85Kr using a two-bed breakthrough method. The topic is very interesting and the founding is attractive. However, their materials are already known ones and their results do not provide sufficient scientific insight.

Most of all, the authors did not provide any explanation why SIFSIX-3-Cu is more radioactively stable than the other SIFSIX-3-M materials. They should have provided suggestions or explanations for the reason by using proper calculations or further experimental characterizations.

Secondly, they argued that their materials have high Kr/N2 selectivities than other benchmark adsorbents. But, they did not provide any comparison with other adsorbents. Moreover, I guess many other adsorbents can have good Kr/N2 selectivities due to the differences in the polarizabilities of two molecules. It would have been more meaningful if the radioactive stability of SIFSIX-3-Cu was compared with other benchmark adsorbents.

For the above reasons, it is regrettable that I cannot recommend this paper to be published in a prestige journal like Nature Comm.

Reviewer #2 (Remarks to the Author):

This manuscript explores the possibility of using SIFSIX-based small pore MOFs for Kr separation from fission gas in the presence of Xe, CO2, N2 and O2. The authors identified that SIFSIX-Cu is not only stable enough under radiation, but also has the ability of capture and separation of Kr from the UNF off-gas if a two-bed arrangement is used. The work is important and novel. The results are interesting. The paper is well written. I recommend it be accepted after the authors take care of the following.

It is not immediate clear from the main text if the PXRD patterns shown in Figure 2 are as-made or activated samples. But the SI indicates that the authors only examined as-made materials. I strongly believe that the authors should irradiate activated MOFs and investigate their stability as the phase behavior of as-made and activated MOFs can be very different. It is the activated MOFs that are used for adsorption. A recent paper (J. Phys. Chem. C 2019, 123, 17798−17807) shows that the metal environment in as-made and activated SIFSIX-Zn can be very different, which should be cited.

I was also wondering if they looked at those unknown phases to see if they still adsorb Kr.

Is there any evidence backing up the very last sentence of Results and Discussion section suggesting Kr adsorption is due to SiF62- pillars?

Reviewer #3 (Remarks to the Author):

Management of spent nuclear fuel is one of the important challenges in any nuclear industry because of high radio-toxicity associated with it. Various physico-chemical processes are framed for the extraction of valuable elements from nuclear wastes and containment of radioactive gases. However, all the chemicals or materials to be used for these processes must be radiation-resistant for them to work efficiently. Chosen chemicals or materials designed for extraction of valuable elements either through liquid-liquid extraction processes or trapping of various fission gasses by porous materials (e.g. Metal-organic frameworks) will not be of any use if the designed materials are not stable in presence of high radiation dose. Therefore, designing radiation-resistant efficient materials is very

important for management of nuclear wastes. Among various fission gases, managing radioactive xenon (Xe) and krypton (Kr) is a great challenge because of inert nature of the noble gases, Xe and Kr. The present communication deals with performance evaluation of a family of ultra-micro-porous metal-organic framework materials, SIFSIX-3-M (M = Fe, Co, Ni, Cu, Zn) for Kr removal from used nuclear fuel. Among all the materials considered in this work, SIFSIX-3-Cu has been found to be the most stable one when subjected to gamma radiation. The most important outcome of the present study is to selectively adsorb Kr gas in the second bed. In my opinion, the proposed radiation-resistant material, SIFSIX-3-Cu, for the purpose of selective adsorption of Xe and Kr seems to be quite efficient, which has been thoroughly investigated through various techniques including computational methods. The work presented in the manuscript is novel for two reasons: (1) selective adsorption of Kr and (2) Evaluation study of irradiation stability of MOF, and subsequent establishment of SIFSIX-3-Cu as a radiation-resistant MOF. It may be accepted for publication in Nature Communications after following points are addressed appropriately:

Is it possible to do two-bed breakthrough experiments with different loadings of noble gases (other than 400 ppm of Xe and 40 ppm of Kr), keeping the Xe/Kr ratio same? This study will provide new insights.

Irradiation induced phase change has been mentioned and the results are reported in the supplementary information for various radiation doses. One of the figures from the supplementary information (particularly the Figure S15 corresponding to 50 kGy, SIFSIX-3-Cu) may be moved to the main manuscript. The possibility of fragmentation through radiation degradation of a material cannot be ruled out; however, no such comment is made in the manuscript.

Grimme's D3 semiempirical method (available in VASP) is more accurate and should be used instead of D2 method. VASP version is not mentioned!

In the supplementary information: I fail to understand the phrase, "the Xe•••F distances for the annealed position were measured to be 1.94, 1.96, 1.98, and 2.00 Å, while distances of 2.14, 2.18, 2.22, and 2.25 Å were measured for the Kr•••F interaction." What do you want to convey with the shorter Xe---F distances ? Also how Xe---F distance is shorter than the Kr---F distance?

It has been demonstrated that a combination of weak intermolecular interactions at low loadings and strong guest–guest interactions at high loadings leading to self-aggregation of guest molecules within confined space are responsible for adsorption of gasses in MFM-300 metal-organic frameworks. This confinement induced adsorption is a comparatively new strategy and related papers may be cited : . Zhang et al, "Confinement of Iodine Molecules into Triple-Helical Chains within Robust Metal–Organic Frameworks", J. Am. Chem. Soc. 2017, 139, 16289-16296; Srinivasu et al, "Confinement-Directed Adsorption of Noble Gases (Xe/Kr) in MFM-300(M)-Based Metal−Organic Framework Materials" J. Phys. Chem. C 2019, 123, 27531-27541.

Reviewer #1:

The authors found that among the series of SIFSIX-3 MOFs, SIFSIX-3-Cu is good for 85Kr removal from nuclear reprocessing plants because it is radioactively stable and can selectively separate and store 85Kr using a two-bed breakthrough method. The topic is very interesting and the founding is attractive. However, their materials are already known ones and their results do not provide sufficient scientific insight.

Most of all, the authors did not provide any explanation why SIFSIX-3-Cu is more radioactively stable than the other SIFSIX-3-M materials. They should have provided suggestions or explanations for the reason by using proper calculations or further experimental characterizations.

Cu-N bonds are stronger than those of other M-N analogues. This is supported by shorter Cu-N bond distance of 1.9 Å in the SIFSIX-3-Cu compared to other M-N analogues (2.1 Å). The stronger Cu bonds lead to a more robust structure. Also, it was reported that the SIFSIX-3-Cu has a slightly smaller unit cell of 378 versus 388$Å^3$ for SIFSIX-3-Zn, where the authors attributed this observation to the relatively stronger bonding between the Cu(II) and the pyrazine.

Secondly, they argued that their materials have high Kr/N2 selectivities than other benchmark adsorbents. But, they did not provide any comparison with other adsorbents. Moreover, I guess many other adsorbents can have good Kr/N2 selectivities due to the differences in the polarizabilities of two molecules. It would have been more meaningful if the radioactive stability of SIFSIX-3-Cu was compared with other benchmark adsorbents.

The choice of the right material for the ^{85}Kr separation from nuclear reprocessing plants is based on several criteria: (1) Preferential adsorption of Xe and CO_2 over Kr so these two gases can be separated in the first bed, and (2) Preferential adsorption of Kr over N_2 and O_2 so the Kr can be adsorbed in the second bed in more pure form with minimum waste volume. If the material fulfills these two criteria, then it should be qualified for the radiation stability study. If the material does not achieve these two criteria, it is trivial to examine their radiation stability because they did not fulfill the main purpose of the Kr removal presented herein, which is the reduction of waste volume and Kr separation in more pure form with minimal amounts of other competing gases. The benchmark materials, SBMOF-1, Ni-MOF-74 and Ag mordenite, showed remarkable performance for Xe/Kr separation, however, they are not suitable for the ^{85}Kr separation from spent fuel using the two-bed technique. Ni-MOF-74 and Ag mordenite were found to have poor Kr/N_2 selectivity, while, SBMOF-1[24] showed low Kr/CO_2 which prohibit the separation of the Kr in pure form.

Figure S21 in the SI demonstrated the low Kr/N_2 selectivity of the Ni-MOF-74. The separation data of Ag mordenite and SBMOF-1 are reported in the literature and the references are cited in the manuscript.

We thank the reviewer for this excellent suggestion to investigate the radiation stability of the benchmark materials. We are currently carrying out a more comprehensive study of the radiation stability of various materials, which will be included in a future paper.

For the above reasons, it is regrettable that I cannot recommend this paper to be published in a prestige journal like Nature Comm.

Reviewer #2:

This manuscript explores the possibility of using SIFSIX-based small pore MOFs for Kr separation from fission gas in the presence of Xe, CO2, N2 and O2. The authors identified that SIFSIX-Cu is not only stable enough under radiation, but also has the ability of capture and separation of Kr from the UNF off-gas if a two-bed arrangement is used. The work is important and novel. The results are interesting. The paper is well written. I recommend it be accepted after the authors take care of the following.

It is not immediate clear from the main text if the PXRD patterns shown in Figure 2 are as-made or activated samples. But the SI indicates that the authors only examined as-made materials. I strongly believe that the authors should irradiate activated MOFs and investigate their stability as the phase behavior of as-made and activated MOFs can be very different. It is the activated MOFs that are used for adsorption. A recent paper (J. Phys. Chem. C 2019, 123, 17798−17807) shows that the metal environment in as-made and activated SIFSIX-Zn can be very different, which should be cited.

The SIFSIX-3-Cu has the same radiation stability either at the as-synthesized form or the activated form. Further study of the stability of the material under Beta radiation was performed on the activated SIFSIX-3-Cu sample and the PXRD pattern was compared with the simulated pattern in Figure 2f.

I was also wondering if they looked at those unknown phases to see if they still adsorb Kr.

We believe any destruction or degradation of the SIFSIX-3-M materials will lead to non-porous structures from the crystallographic point of view since SIFSIX-3-M is a ***pcu*** network and has only one-dimensional channel. Even if only the M-F bond gets broken to go from 3D to 2D structure the SiF_6 pillar has to travel to the channel, which will block the pore especially the material has a very narrow pore of 3.6 Å.

Is there any evidence backing up the very last sentence of Results and Discussion section suggesting Kr adsorption is due to SiF62- pillars?

Figures 3(c) and S23 show that the modeled binding site for Kr in **SIFSIX-3-Cu** is between four neighboring SiF_6^{2-} pillars within the square grid.

Furthermore, we performed periodic density functional theory (DFT) calculations for Xe, Kr, CO_2, N_2, and O_2 within **SIFSIX-3-Cu** to determine the optimal binding site for these gases in the material and subsequently compute the adsorption energies (ΔE). The details for executing these calculations are now added to the "Modeling studies in SIFSIX-3-Cu" section in the SI. The optimized position for all adsorbates in the MOF was discovered to be between four adjacent SiF_6^{2-} pillars. Figures illustrating these have been added to the SI (now Figures S24-S28). In addition, the following sentence is now listed as the very last sentence of the "Results and Discussion" section of the manuscript:

"Indeed, modeling studies revealed that the adsorbate localizes between four neighboring SiF_6^{2-} pillars within the small pores in this class of materials."

Additionally, we now provide the calculated ΔE values for all adsorbates in **SIFSIX-3-Cu** in Table S1. The following trend can be observed in the MOF–adsorbate interaction strength on the basis of these values: CO_2 > Xe > Kr > N_2 > O_2. This is fully consistent with the trends in the gas uptakes within the experimental adsorption isotherms displayed in Figure 3(a). Moreover, these results support the experimental findings/conclusions that **SIFSIX-3-Cu** preferentially adsorb CO_2 and Xe over Kr and Kr over N_2 and O_2. The following sentence has been added to the end of the third paragraph of the "Results and Discussion" section of the manuscript to indicate that such calculations support the experimental results:

"Furthermore, periodic density functional theory (DFT) calculations confirm that SIFSIX-3-Cu is selective for CO_2 and Xe over Kr and Kr over N_2 and O_2 on the basis of the calculated adsorption energies in the material (Table S1)."

Reviewer #3:

Management of spent nuclear fuel is one of the important challenges in any nuclear industry because of high radio-toxicity associated with it. Various physico-chemical processes are framed for the extraction of valuable elements from nuclear wastes and containment of radioactive gases. However, all the chemicals or materials to be used for these processes must be radiation-resistant for them to work efficiently. Chosen chemicals or materials designed for extraction of valuable elements either through liquid-liquid extraction processes or trapping of various fission gasses by porous materials (e.g. Metal-organic frameworks) will not be of any use if the designed materials are not stable in presence of high radiation dose. Therefore, designing radiation-resistant efficient materials is very important for management of nuclear wastes. Among various fission gases, managing radioactive xenon (Xe) and krypton (Kr) is a great challenge because of inert nature of the noble gases, Xe and Kr. The present communication deals with performance evaluation of a family of ultra-micro-porous metal-organic framework materials, SIFSIX-3-M (M = Fe, Co, Ni, Cu, Zn) for Kr removal from used nuclear fuel. Among all the materials considered in this work, SIFSIX-3-Cu has been found to be the most stable one

when subjected to gamma radiation. The most important outcome of the present study is to selectively adsorb Kr gas in the second bed. In my opinion, the proposed radiation-resistant material, SIFSIX-3-Cu, for the purpose of selective adsorption of Xe and Kr seems to be quite efficient, which has been thoroughly investigated through various techniques including computational methods. The work presented in the manuscript is novel for two reasons: (1) selective adsorption of Kr and (2) Evaluation study of irradiation stability of MOF, and subsequent establishment of SIFSIX-3-Cu as a radiation-resistant MOF. It may be accepted for publication in Nature Communications after following points are addressed appropriately:

Is it possible to do two-bed breakthrough experiments with different loadings of noble gases (other than 400 ppm of Xe and 40 ppm of Kr), keeping the Xe/Kr ratio same? This study will provide new insights.

The choice of this composition is based on the actual composition of UNF off-gas. However, this is an insightful comment and will consider these measurements in future work.

We agree with reviewer, keeping noble gas concentration the same and varying other gas composition provide new insights but these experiments are beyond the scope of this work. However, we will follow up with another paper focused on reviewer suggestion and role of particle size and shape affect the noble gas selectivity.

Irradiation induced phase change has been mentioned and the results are reported in the supplementary information for various radiation doses. One of the figures from the supplementary information (particularly the Figure S15 corresponding to 50 kGy, SIFSIX-3-Cu) may be moved to the main manuscript. The possibility of fragmentation through radiation degradation of a material cannot be ruled out; however, no such comment is made in the manuscript.

Figure 2f was added to the manuscript that also demonstrated the stability of the SIFSIX-3-Cu under beta radiation and the suggested comment was discussed in the manuscript.

Grimme's D3 semiempirical method (available in VASP) is more accurate and should be used instead of D2 method. VASP version is not mentioned!

We optimized the unit cell of **SIFSIX-3-Cu** using the DFT-D3 method, but surprisingly found that the energy of the resulting crystal structure is higher than that obtained using the DFT-D2 method (–705.5814605 vs –706.2898731 eV). Because the DFT-D2 method resulted in a crystal structure that was lower in energy, we decided to use this structure for the classical simulations. This is now summarized within the second paragraph of the "Modeling studies in SIFSIX-3-Cu" section in the SI.

The version of VASP that was utilized is 5.4.4; this is now mentioned in the aforementioned section in the SI.

In the supplementary information: I fail to understand the phrase, "the Xe•••F distances for the annealed position were measured to be 1.94, 1.96, 1.98, and 2.00 Å, while distances of 2.14, 2.18, 2.22, and 2.25 Å were measured for the Kr•••F interaction." What do you want to convey with the shorter Xe---F distances ? Also how Xe---F distance is shorter than the Kr---F distance?

If considering the distances between the center-of-mass (COM) of the equatorial F atoms and the COM of both Xe and Kr, then the measured distances would be nearly identical between the adsorbates. For the record, when measuring between the two COMs, the Xe···F distances are 3.44, 3.46, 3.48, 3.50 Å, while those for Kr···F are 3.44, 3.48, 3.52, and 3.55 Å. However, if these distances are subtracted by both the atomic radius of F (0.42 Å) and the atomic radius for the noble gas (Xe = 1.08 Å, Kr = 0.88 Å), then distances of 1.94, 1.96, 1.98, and 2.00 Å are obtained for the Xe···F interaction, while distances of 2.14, 2.18, 2.22, and 2.25 Å are obtained for Kr···F. Since Xe exhibits a larger atomic radius than Kr, the distance between the *surface* of the atom to that of an equatorial F atom is shorter than the corresponding distance for Kr···F.

It is well-known in the MOF literature that shorter adsorbent–adsorbate distances corresponds to stronger interactions between the adsorbent and the adsorbate. By mentioning that the Xe···F interaction distances are shorter than those for Kr···F, we want to imply that the interaction between **SIFSIX-3-Cu** and Xe is stronger compared to that between the MOF and Kr. This is consistent with experimental findings that **SIFSIX-3-Cu** displays higher affinity toward Xe than Kr.

It has been demonstrated that a combination of weak intermolecular interactions at low loadings and strong guest–guest interactions at high loadings leading to self-aggregation of guest molecules within confined space are responsible for adsorption of gasses in MFM-300 metal-organic frameworks. This confinement induced adsorption is a comparatively new strategy and related papers may be cited : . Zhang et al, "Confinement of Iodine Molecules into Triple-Helical Chains within Robust Metal–Organic Frameworks", J. Am. Chem. Soc. 2017, 139, 16289-16296; Srinivasu et al, "Confinement-Directed Adsorption of Noble Gases (Xe/Kr) in MFM-300(M)-Based Metal−Organic Framework Materials" J. Phys. Chem. C 2019, 123, 27531-27541.

The suggested paper *" J. Phys. Chem. C 2019, 123, 27531-27541"* is cited in the manuscript. Thanks for the suggestion.

Reviewers' comments:

Reviewer #1 (Remarks to the Author):

I still think that major issues raised by the reviewer have not been fully addressed. I recommend the publication if the following points are clearly addressed.

The authors attributed the observed radioactive stability of SIFSIX-3-Cu to the stronger Cu-N bonds than other M-N analogues by mentioning the shorter Cu-N bond distance (1.9 Å) than the other the M-N bond distances (2.1 Å). From the PXRD measurements after the gamma radiation at different intensities, the radioactive stabilities of SIFSIX-M show the following order: Cu > Co > Fe > Zn and Ni. Could this order be explained by the bond distances? The authors should clearly explain this.

The authors argued that they chose the right material for the 85Kr separation from nuclear reprocessing plants based on two criteria: (1) Preferential adsorption of Xe and CO2 over Kr so these two gases can be separated in the first bed, and (2) Preferential adsorption of Kr over N2 and O2 so that Kr can be adsorbed in the second bed. However, I do not understand why the same adsorbent material should be used for both beds. I think two different adsorbent materials can be used for each bed. Since SBMOF-1, Ni-MOF-74 and Ag mordenite showed remarkable performance for Xe/Kr separation, these materials can be used for the first bed. Therefore, it would be more meaningful if the radioactive stability of these benchmark adsorbents are also compared.

Although the authors cited the references including the Kr/N2 separation data of Ag mordenite and SBMOF-1, these should be shown in the supporting information for the comparisons.

I found the following paper: "Adsorptive separation of xenon/krypton mixtures using a zirconium-based metal-organic framework with high hydrothermal and radioactive stabilities, Journal of Hazardous Materials 320 (2016) 513–520." This paper also studied radioactive stability of a zirconium-based MOF for Xe/Kr separation. I guess the authors may have not noticed this paper. It should be properly referenced.

Page 8, line 165: "SBMOF-1 showed low Kr/CO2 which prohibit the separation of the Kr in pure form."
--> Is low Kr/CO2 correct? It could be low Kr/O2.

Reviewer #2 (Remarks to the Author):

The authors addressed most of the issues raised by three reviewers reasonably well. Thus, I am supportive of acceptance. However, both reviewers 2 and 3 suggested several references to be included and it is disappointing to see only one was cited. I hope they will all be included in the final version.

Reviewer #3 (Remarks to the Author):

Authors have suitably revised the manuscript. By and large all the comments are addressed successfully. I recommend the manuscript to be accepted as is.

Reviewers' comments:

Reviewer #1 (Remarks to the Author):

I still think that major issues raised by the reviewer have not been fully addressed. I recommend the publication if the following points are clearly addressed.

The authors attributed the observed radioactive stability of SIFSIX-3-Cu to the stronger Cu-N bonds than other M-N analogues by mentioning the shorter Cu-N bond distance (1.9 Å) than the other the M-N bond distances (2.1 Å). From the PXRD measurements after the gamma radiation at different intensities, the radioactive stabilities of SIFSIX-M show the following order: Cu > Co > Fe > Zn and Ni. Could this order be explained by the bond distances? The authors should clearly explain this.

We thank the reviewer for highlighting this point. We are aware that more in-depth study is required to better understand the mechanism of radiation absorption by the framework. This will be part of our future studies as it depends on many factors and it is out of the scope of the current work. The scope of the paper is more toward application more than theoretical investigation. There are only few reported studies for radiation stability of MOFs and we believe more studies need to be done at this area. For example, we tried to use the NIST XCOM: Photon Cross Sections Database which is mainly looking to the metal center as the main factor for stability. However, the results did not support our data, presumably, because there are many factors that can contribute to the radiation stability not only the metal centers since MOFs are composed of metal, organic and/or inorganic linkers with M-O/M-N/M-F bonds. We believe that the radiation stability of the MOF framework can depend on many factors such as chemical bond strength, understanding which part of the framework absorbs the energy and where this energy goes. We think better model need to be created and more careful calculation is needed

Therefore, we believe that the current explanation of the shorter Cu-N bond distance compared to the other analogues is enough at this stage to elucidate why the SIFSIX-3-Cu is the more radiation-stable material and thereby the best analogue for the application presented herein. SIFSIX-3-Cu has shorter M-N bond of 1.9 Å compared to other analogues which show very small differences in M-N bond distance. According to CCDC, Cu tends to bond with N donor ligand more readily compared to Co, Fe, Zn and Ni. Therefore, bond strength could be the main factor behind the stability of SIFSIX-Cu framework which reaches up to 50 kGy, while other materials demonstrate much lower stability that reaches to only 10 kGy in case of Co. The bond distance in the other analogues are around the same 2.15±0.04 Å. We believe there are other factors controlling the stability of the other analogues and we are currently working on making a model to better understand the radiation absorption by the framework.

The authors argued that they chose the right material for the 85Kr separation from nuclear reprocessing plants based on two criteria: (1) Preferential adsorption of Xe and CO2 over Kr so these two gases can be separated in the first bed, and (2) Preferential adsorption of Kr over N2 and O2 so that Kr can be adsorbed in the second bed. However, I do not understand why the

same adsorbent material should be used for both beds. I think two different adsorbent materials can be used for each bed. Since SBMOF-1, Ni-MOF-74 and Ag mordenite showed remarkable performance for Xe/Kr separation, these materials can be used for the first bed. Therefore, it would be more meaningful if the radioactive stability of these benchmark adsorbents are also compared.

We are currently working on the radiation stability of several MOFs including the aforementioned MOFs by the reviewer and theoretical study on the mechanism of radiation absorption by the MOF structure. This data will be included in a follow-up paper.

Although the authors cited the references including the Kr/N2 separation data of Ag mordenite and SBMOF-1, these should be shown in the supporting information for the comparisons.

The data for the SBMOF-1 and Ag mordenite were added in **Figure S22** and **Figure S30** in the SI.

I found the following paper: "Adsorptive separation of xenon/krypton mixtures using a zirconium-based metal-organic framework with high hydrothermal and radioactive stabilities, Journal of Hazardous Materials 320 (2016) 513–520." This paper also studied radioactive stability of a zirconium-based MOF for Xe/Kr separation. I guess the authors may have not noticed this paper. It should be properly referenced.

The paper is properly discussed and cited in the main manuscript (***Page 3***).

Page 8, line 165: "SBMOF-1 showed low Kr/CO2 which prohibit the separation of the Kr in pure form." --> *Is low Kr/CO2 correct? It could be low Kr/O2.*

This is not a mistake. As shown in **Figure S22** in the SI, SBMOF-1 shows very low Kr/CO2 selectivity which means that the CO2 cannot be separated from Kr.

Reviewer #2 (Remarks to the Author):

The authors addressed most of the issues raised by three reviewers reasonably well. Thus, I am supportive of acceptance. However, both reviewers 2 and 3 suggested several references to be included and it is disappointing to see only one was cited. I hope they will all be included in the final version.

All the references suggested by reviewers have been cited. Thank you for your kind review of our manuscript.

Reviewer #3 (Remarks to the Author):

Authors have suitably revised the manuscript. By and large all the comments are addressed successfully. I recommend the manuscript to be accepted as is.

Thank you for your kind recommendation that our work be accepted to Nature Communications.

REVIEWERS' COMMENTS:

Reviewer #1 (Remarks to the Author):

Although some issues related to the mechanism of radiation absorption have not been fully addressed, I think it is now publishable since most of the comments from the reviewers have been answered and revised.
